# Quantifying dispersal between marine protected areas by a highly mobile species, the bottlenose dolphin, *Tursiops truncatus*

**DOI:** 10.1002/ece3.4343

**Published:** 2018-08-23

**Authors:** Milaja Nykänen, Eileen Dillane, Anneli Englund, Andrew D. Foote, Simon N. Ingram, Marie Louis, Luca Mirimin, Machiel Oudejans, Emer Rogan

**Affiliations:** ^1^ School of Biological, Earth and Environmental Sciences University College Cork Cork Ireland; ^2^ School of Biological Sciences Molecular Ecology Fisheries Genetics Lab Bangor University Bangor UK; ^3^ School of Biological and Marine Sciences Plymouth University Plymouth UK; ^4^ Centre d'Etudes Biologiques de Chizé UMR 7372 CNRS‐Université de La Rochelle La Rochelle France; ^5^ Observatoire Pelagis UMS 3462 CNRS‐Université de La Rochelle La Rochelle France; ^6^ Scottish Oceans Institute University of St Andrews St Andrews UK; ^7^ Department of Natural Sciences School of Science and Computing Galway‐Mayo Institute of Technology Marine and Freshwater Research Centre Galway Ireland; ^8^ Kelp Marine Research Hoorn The Netherlands

**Keywords:** bottlenose dolphins, connectivity, MPAs, photo‐identification, population structure

## Abstract

The functioning of marine protected areas (MPAs) designated for marine megafauna has been criticized due to the high mobility and dispersal potential of these taxa. However, dispersal within a network of small MPAs can be beneficial as connectivity can result in increased effective population size, maintain genetic diversity, and increase robustness to ecological and environmental changes making populations less susceptible to stochastic genetic and demographic effects (i.e., Allee effect). Here, we use both genetic and photo‐identification methods to quantify gene flow and demographic dispersal between MPAs of a highly mobile marine mammal, the bottlenose dolphin *Tursiops truncatus*. We identify three populations in the waters of western Ireland, two of which have largely nonoverlapping core coastal home ranges and are each strongly spatially associated with specific MPAs. We find high site fidelity of individuals within each of these two coastal populations to their respective MPA. We also find low levels of demographic dispersal between the populations, but it remains unclear whether any new gametes are exchanged between populations through these migrants (genetic dispersal). The population sampled in the Shannon Estuary has a low estimated effective population size and appears to be genetically isolated. The second coastal population, sampled outside of the Shannon, may be demographically and genetically connected to other coastal subpopulations around the coastal waters of the UK. We therefore recommend that the methods applied here should be used on a broader geographically sampled dataset to better assess this connectivity.

## 1 INTRODUCTION

The conservation and management of wild animal populations are often achieved through designation of protected areas that are thought to represent important habitats for foraging, breeding, and other fitness‐related activities (Palumbi, 2001; Reeves, 2000). Demographic connectivity, defined as the linking together of local fragmented populations through the dispersal of individuals as larvae, juveniles, or adults (Sale et al., 2005), is an important factor to consider when designating marine protected areas (MPAs), as it has implications for the persistence of metapopulations (reviewed in Botsford et al., 2009). For example, in many marine fish species, larval dispersal and population connectivity determine whether a MPA (or a network of MPAs) contributes to the overall survival and reproduction of the species, thus maintaining sustainable population sizes (Burgess et al., 2014). Dispersal is thus a key variable that conservation biologists need to quantify and consider in order to assess the effectiveness of protected areas (Reeves, 2000). This is particularly relevant in highly mobile and wide‐ranging marine species, whose management provision is often restricted to small fixed areas of protection and for which the low cost of movement can facilitate long‐range dispersal (reviewed in Forcada, 2009). High levels of mobility can result in substantial gene flow and the homogenization of genetic diversity across a geographic range (Ryman, Lagercrantz, Andersson, Chakraborty, & Rosenberg, 1984; Winkelmann et al., 2013). However, whilst in most marine fish metapopulations dispersal during the larval stage facilitates greater connectivity among habitat patches and reduces the risk of local extinctions (Burgess et al., 2014), marine mammals typically have much lower reproductive rates and their offspring can exhibit a high degree of natal philopatry (Amos, Schlotterer, & Tautz, 1993; Baird, 2000; Sellas, Wells, & Rosel, 2005). This can lead to small isolated populations and a system that is sensitive to changes in environmental conditions, ecological factors, or anthropogenic disturbance.

Lowe and Allendorf (2010) distinguished demographic connectivity from genetic connectivity by defining the former as the relative contribution of net immigration and local recruitment to the population growth rate, and the latter as the degree to which evolutionary processes within (sub)populations are affected by gene flow. Population genetic approaches may provide a tool to measure and quantify the rate and scale of dispersal (i.e., migration) when it is not feasible to assess the movement of individuals by nongenetic capture–recapture methods (Gagnaire et al., 2015). However, when combined together, genetic and nongenetic methods are highly complementary and can provide invaluable information for management of populations. Photo‐identification is a cost‐effective technique commonly used by marine mammal researchers to identify individuals of several species using the unique natural markings on their body and thus enabling, for example, the estimation of their distribution, association patterns, or abundance via capture–recapture methods (see review by Würsig & Jefferson, 1990). If natural markings cannot be used because of insufficient individual variation, molecular genotyping may provide a usable alternative to photo‐identification methods in estimating animal movements (see Palsbøll et al., 1997). Here, both these approaches were applied to quantify the demographic and genetic connectivity between marine protected areas designated for bottlenose dolphins in an area in the north‐east Atlantic.

Bottlenose dolphins are widely distributed, being found in the Atlantic, Indian, and Pacific oceans (Leatherwood & Reeves, 1990). Throughout much of its range, the common bottlenose dolphin (*Tursiops truncatus*) exhibits hierarchical population structure, with the greatest divergence found between pelagic and coastal populations (Curry & Smith, 1998; Hoelzel, Potter, & Best, 1998; Louis, Fontaine et al., 2014; Louis, Viricel et al., 2014; Lowther‐Thieleking, Archer, Lang, & Weller, 2015). Genetic differentiation is often correlated with ecological and/or morphological differences (Hersh & Duffield, 1990; Hoelzel et al., 1998; Louis, Viricel et al., 2014; Natoli, Peddemors, & Hoelzel, 2004). Further fine‐scale structuring has been found among coastal populations in several locations (Baird et al., 2009; Caballero et al., 2012; Fernández et al., 2011; Gaspari et al., 2013, 2015; Louis, Fontaine et al., 2014; Louis, Viricel et al., 2014; Martien, Baird, Hedrick, & Webster, 2011; Martinho, Pereira, Brito, Gaspar, & Carvalho, 2014; Mirimin et al., 2011; Natoli, Birkun, Aguilar, Lopez, & Hoelzel, 2005; Parsons, Noble, Reid, & Thompson, 2002; Parsons et al., 2006; Rosel, Hansen, & Hohn, 2009). The driving force(s) behind fine‐scale population structuring among coastal populations of bottlenose dolphins are not fully resolved, but have been suggested to include isolation following a historical founding event; habitat preferences; differences in social structure and site fidelity; learned foraging specializations; natal philopatry; limited dispersal of both sexes; and habitat discontinuity linked to prey availability (Gaspari et al., 2015; Krützen, Barre, Connor, Mann, & Scherwin, 2004; Krützen, Scherwin, Berggren, & Gales, 2004; Louis, Fontaine et al., 2014; Louis, Viricel et al., 2014; Martien et al., 2011; Natoli et al., 2005; Parsons et al., 2006; Rosel et al., 2009).

**Figure 1:**
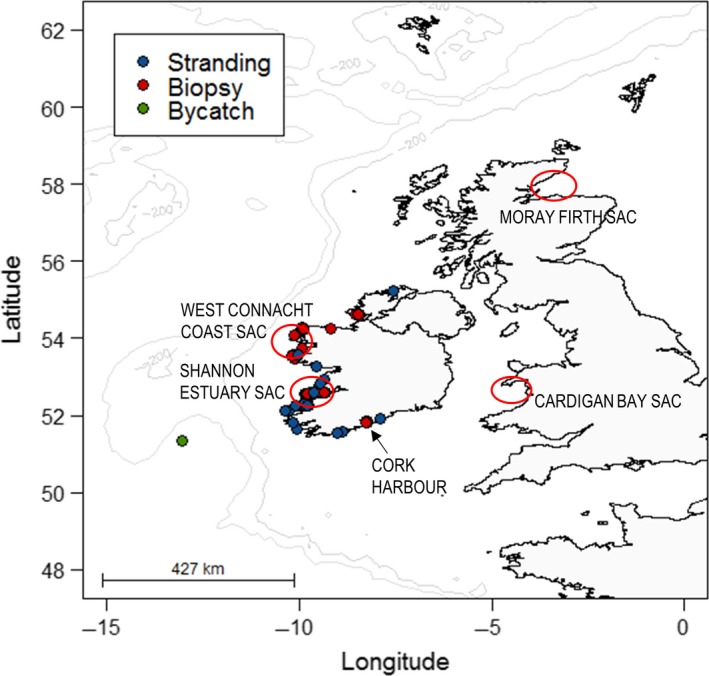
GPS locations of bottlenose dolphin samples collected and used throughout this study and approximate locations of Special Areas of Conservation (SACs) around the British Isles (areas circled). Samples include coastal biopsies of free‐living dolphins (*n* = 71), samples collected from dead‐stranded animals (*n* = 25), and one sample from a bycaught animal. Note that some sampling locations indicated by the circles overlap due to the scale of the map

Common bottlenose dolphins are listed in Annex II of the European Union's Habitats Directive requiring the member states to designate Special Areas of Conservation (SACs) as part of an overall European strategy (Natura 2000) to maintain or restore the species at “favourable conservation status.” Therefore, SACs (or Natura 2000 sites) have been designated in the coastal waters of several areas in EU Member States. Around the British Isles, such SACs are located in Moray Firth (Scotland), Cardigan Bay (Wales), and two areas on the west coast of Ireland, the Shannon Estuary and in western parts of Counties Galway and Mayo (West Connacht Coast) (see Figure 1). However, it is unclear how much connectivity (genetic or demographic) there is between the different groups of bottlenose dolphins inhabiting these areas.

Bottlenose dolphins using the Shannon Estuary SAC have been found to be genetically differentiated from another population inhabiting the coastal waters of counties Galway and Mayo (Mirimin et al., 2011). However, these findings were based on a limited number of samples collected in a relatively small area (ranging about 70 km along the Galway/Mayo coastline) and it is not known whether additional fine‐scale structuring exists. Photo‐identification studies of dolphins using the Shannon Estuary SAC suggest that these individuals have a high degree of site fidelity (e.g., Englund, Ingram, & Rogan, 2008; Ingram & Rogan, 2003); however, the extent of the range of dolphins using Ireland's coastal waters is not yet fully understood. Previous research has shown that at least some of these coastal animals move over great distances (Cheney et al., 2013; Ingram, Englund, & Rogan, 2001, 2003; O'Brien et al., 2009; Oudejans, Ingram, Englund, & Rogan, 2010; Robinson et al., 2012), which could indicate some potential for genetic connectivity between adjacent subpopulations using neighboring coastal SACs, but this has not previously been demonstrated or quantified.

Genetic clustering and kinship‐based methods are used here to reexamine the population structure in Irish waters using a larger dataset supplemented with samples collected from a wider coastal area. The contribution of demographic and genetic dispersal to the connectivity between neighboring SACs within Irish waters is quantified using a combination of photo‐identification and genetic techniques. In addition, the role of possible drivers for population structuring, including social structure, relatedness, site fidelity, and sex‐biased dispersal, are examined. The findings are discussed in the context of conservation and management.

## 2 MATERIALS AND METHODS

### 2.1 Photo‐identification surveys and photograph selection

**Figure 2:**
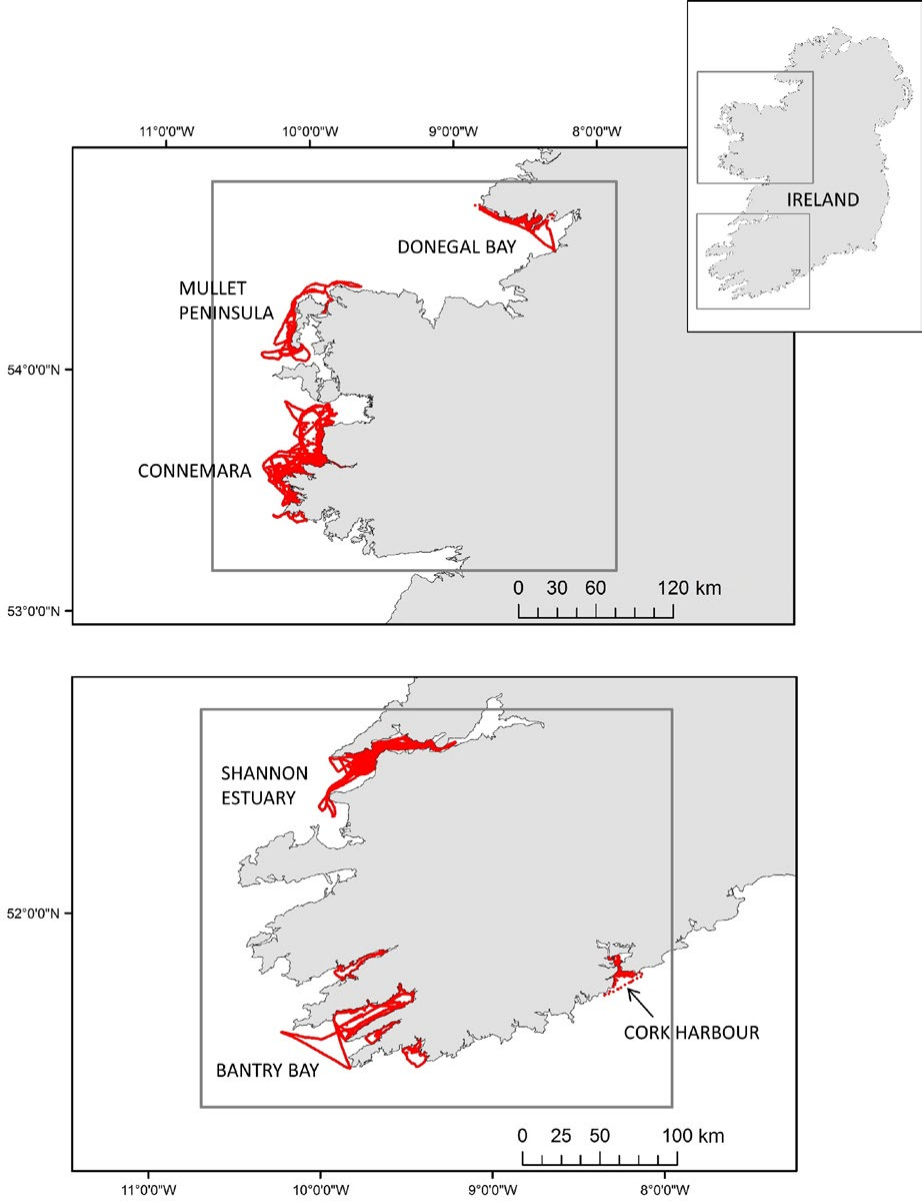
GPS tracks recorded during boat surveys for bottlenose dolphins on the West coast of Ireland

**Figure 3:**
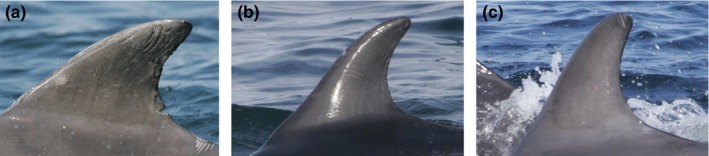
Examples of bottlenose dolphin fins showing the three grades of mark severity used in photograph analysis. Each dolphin was graded from one to three as follows: (a) grade M1 marks, consisting of significant fin damage or deep scarring that were considered permanent; (b) grade M2 marking that consist of deep tooth rakes and lesions, with only minor cuts present; (c) fin with grade M3 marks, having only superficial rakes and lesions. Grades M1 and M2 are considered to last many years, enabling long‐term identification of these dolphins. In contrast, “superficial” markings (grade M3), such as tooth rakes, may fade and heal within a relatively short period of time and interannual resighting probabilities of these animals are likely to be reduced

Boat‐based photo‐identification surveys were conducted within the Lower River Shannon SAC, Ireland, every year between 1996 to 2008 with the exception of 2004, and in other coastal areas of Ireland (including the West Connacht Coast SAC), in 2001–2005, 2007–2010, and 2013–2014 (Figures 1 and 2). These surveys were mostly conducted during the summer months (May–September), however, some were done in autumn or winter (see Supporting information Table S1 in dryad for the survey information). A bottlenose dolphin “group” was defined as all dolphins within a 100 m radius of each other as per Irvine, Scott, Wells, and Kaufmann (1981) and hereafter “encounters” refer to periods of data collection whilst with dolphin groups. Best effort was made to photograph every individual in the group, and photograph identification of bottlenose dolphins’ dorsal fins was examined. For each encounter, the best quality photograph was chosen of each identifiable dolphin and the quality of the photograph was graded from 1 to 4 (1 being the highest quality, 4 being the lowest, see Supporting information Appendix S1) with no consideration concerning the degree of marking of the individual. Each photographed individual was then assigned one of three grades of mark severity (Figure 3), and visually matched against the full catalogue of dolphins photographed during previous encounters.

### 2.2 Skin tissue sample collection and analysis

The dataset comprising of altogether 97 unique samples included 85 samples already genotyped by Mirimin et al. (2011). This set of 85 genotypes included 45 skin tissue samples collected from animals in the Shannon Estuary SAC in 2005 and 2007, four samples from animals encountered in Cork Harbour in 2008 and 12 samples collected from animals ranging in coastal waters of Galway and Mayo (part of West Connacht Coast SAC) during 2009 (Figure 1). The previously genotyped dataset also included samples collected from 23 individuals stranded along the west coast of Ireland, including two dolphins found dead within the Shannon Estuary, between 1993 and 2009. This dataset was supplemented by ten skin biopsies collected from free‐ranging animals in coastal waters of Co. Mayo and Co. Donegal during 2013–2014, a sample from a dolphin that stranded in Co. Cork in 2014, and a sample collected from an animal that was bycaught by a fishing vessel on the continental shelf off south‐west of Ireland in 1996. All of the skin biopsy samples in this study were taken using a modified 0.22 caliber rifle (see Krützen et al., 2002) and sampling was carried out during the summer months. The gender of stranded individuals was recorded by inspection of the genital area and reproductive organs, whilst the sex of free‐ranging biopsied individuals was determined by multiplex amplification of sex chromosome‐specific DNA fragments, following the method described in Rosel (2003).

### 2.3 DNA extraction, PCR amplification, and genotyping

DNA was extracted from 12 new skin samples using the DNeasy Blood and Tissue kit from Qiagen. A total of 15 nuclear microsatellite loci (see Supporting information Appendix S2) were amplified following polymerase chain reaction (PCR) conditions described in Mirimin et al. (2011). The amplified products were separated on 6% polyacrylamide gels on a Li‐Cor 4300 DNA analyzer (Li‐Cor Inc, Lincoln, NE, USA) and allele sizes determined by eye in comparison with a 50–530 size standard (Li‐Cor) and allele cocktails from reference samples. These allele cocktails consisted of mixtures of PCR products from four to five individuals previously genotyped for each locus and allowed alleles in this study to be consistently sized across runs and in line with the samples of Mirimin et al. (2011). Due to the possibility that the same individual dolphin may have been unintentionally biopsied more than once, the uniqueness of the new genotypes was confirmed by calculating the percentage of similarity between the samples in program GIMLET 1.3.3. (Valière, 2002). The same program was also used to calculate the probability of identity (PI), which estimates the power of the set of microsatellite markers to differentiate between two distinct individual samples (Waits, Luikart, & Taberlet, 2001). The error rate involved in genotyping had already been estimated as negligible (<0.01%) by Mirimin et al. (2011), therefore, reestimation of the error was not performed for the new samples because of their low number (*n *=* *12).

The 15 microsatellite loci were checked for null alleles, allelic dropout, and stuttering, using MICRO‐CHECKER 2.2.3 (Van Oosterhout, Hutchinson, Wills, & Shipley, 2004) and selecting the Bonferroni‐adjusted 95% confidence interval option with 1,000 simulations. In addition, MICRODROP 1.01 (Wang, Schroeder, & Rosenberg, 2012) was used to further check for allelic dropout due to low DNA concentration or poor sample quality. The microsatellite loci were inspected for significant deviations from Hardy–Weinberg equilibrium (HWE) using GENEPOP (Raymond & Rousset, 1995; Rousset, 2008) and linkage equilibrium using ARLEQUIN (Excoffier & Lischer, 2010) with 10,000 iterations and applying sequential Bonferroni corrections. The above analyses were performed considering the whole dataset as a single unit and separately at population level (identified with Bayesian clustering methods, see below).

### 2.4 Individual assignment tests

All samples were included in a cluster analysis using STRUCTURE (Pritchard, Stephens, & Donnelly, 2000). The admixture model was run with correlated allele frequencies without including any prior information on the sampling location. Ten independent runs were carried out for each value of *K* (the number of theoretical populations), with *K* set to vary from 1 to 6, using 1,000,000 Markov Chain Monte Carlo (MCMC) iterations preceded by 1,000,000 burn‐in steps. Convergence of chains (traces of alpha and *F*_ST_ values) was confirmed visually and the consistency of runs was checked by confirming that the variance in estimated ln Pr(*X*|*K*) was smaller within each *K* compared to the variance between the different *K*s, and calculating the average posterior probability for each *K. ∆K,* which has been argued to be a better predictor of the number of populations, was also calculated following Evanno, Regnaut, and Goudet (2005) in STRUCTURE HARVESTER Web version 0.6.94 (Earl & vonHoldt, 2012). Once *K* was determined, each individual was assigned to a cluster based on its maximum membership proportion.

As relatedness between individuals can affect population assignment (i.e., including samples of closely related individuals can lead to artificial structuring of populations (Guinand et al., 2006; Anderson & Dunham, 2008), the relatedness coefficient, *r*, (Queller & Goodnight, 1989) was calculated between all possible dyads within the putative populations identified by the clustering methods using KINGROUP (Konovalov, Manning, & Henshaw, 2004). Then, one member of each dyad with a relatedness coefficient of 0.45 or greater was removed (according to Rosel et al., 2009) and STRUCTURE re‐run with this reduced dataset.

In addition, population structuring was inferred using a discriminant analysis of principal components (DAPC) that clusters individuals together based on genetic similarity to find the most likely number of populations. DAPC does not rely on any population genetic model (i.e., does not assume HWE) and is efficient at detecting hierarchical structure (Jombart, Devillard, & Balloux, 2010). DAPC using the package adegenet (Jombart, 2008) in R (R Core Team 2016)was run following the recommendations in the tutorial (Jombart & Collins 2015), and cluster membership probabilities were calculated for each individual.

A third clustering method was implemented in program TESS (Durand, Chen, & Francois, 2009; Durand, Jay, Gaggiotti, & Francois, 2009) which uses GPS coordinates along with genetic markers to infer population structure; therefore only biopsy samples were used in this analysis as stranded and bycaught individuals had unknown geographic origins. The conditional autoregressive (CAR) model was used with admixture using 20,000 burn‐in followed by 120,000 MCMC steps with the number of clusters, *K*, varying 2–10, with 10 replicates per each run. The most probable number of clusters was selected by plotting Deviance Information Criterion (DIC) values against different values of *K* and by examining individual assignment probability plots. Consistency of the runs was checked by examining the convergence of MCMC chains in TRACER 1.6. (Rambaut, Suchard, Xie, & Drummond, 2014). TESS cannot directly test for *K *=* *1 but we checked this by examining individual assignment probabilities. When the most likely *K* was determined, the run with the lowest DIC was used and individuals were assigned to clusters based on maximum assignment probabilities.

The results from clustering methods when all samples were included (i.e., STRUCTURE and DAPC, see below) were highly consistent in their inference of the most likely number of clusters and the individual assignment probabilities so the dataset was divided into three putative populations, *Coastal Shannon*,* Coastal mobile* and *Pelagic*, for the remaining genetic analyses. There is uncertainty associated with the geographic range of the *Pelagic* population as the samples consist mostly of stranded animals, but based on the fact that these animals have not been photographed in coastal waters coupled with their genetic divergence, and for consistency with previous publications, for example Louis, Viricel et al. (2014), this population is referred to as the *Pelagic* population.

Population differentiation was estimated by calculating pairwise *F*_ST_ (Weir & Cockerham, 1984) and Jost's *D* (Jost, 2008) values using the R package diveRsity (Keenan, McGinnity, Cross, Crozier, & Prodöhl, 2013) between populations identified by STRUCTURE, with the whole and the reduced dataset after the removal of close relatives, and the 95% confidence intervals were obtained using 10,000 bootstrap replicates. Population‐specific *F*_IS_ values, expected and observed heterozygosity, mean number of alleles, and allele richness were also calculated using package diveRsity to examine the level of inbreeding. Heterozygote deficiency and excess in each population was tested using Fisher's method implemented in GENEPOP (Raymond & Rousset, 1995; Rousset, 2008) with 10,000 iterations. As a further check that differentiation was not solely driven by sampling of related individuals or uneven sampling of populations (see Puechmaille, 2016), 10 individuals were randomly selected from each of the two putative coastal populations and the pairwise *F*_ST_ values (with 95% CI) estimated using the R package diveRsity and repeated 10 times. These pairwise values were compared to *F*_ST_ values calculated for two sets of ten individuals randomly drawn from within a single coastal population, *Coastal Shannon* or *Coastal mobile*. To supplement this analysis, the power to detect a significant moderate population differentiation, based on an *F*_ST_ value of ≥0.1 in a sample consisting of the allele frequencies from both coastal populations and using a sample size of ten individuals per “subpopulation” (i.e., *Coastal Shannon* and *Coastal mobile*), was calculated by running 1,000 simulations in POWSIM 4.1 (Ryman & Palm, 2006; see also Ryman et al., 2006; Morin, Martien, & Taylor, 2009).

Sex‐biased dispersal between the three populations identified by clustering methods was tested by comparing assignment indices, relatedness, *F*_ST_ and *F*_IS_ values separately for males and females using 1,000 permutations in FSTAT 2.9.3 (Goudet, 2001). Following Goudet (2001), it was assumed that sex‐biased dispersal within the sampled populations could be detected from gender differences in genetic structuring with the more philopatric sex showing more structure.

### 2.5 Migration rates

Recent migration rates (the proportion of migrants per population) within the last two generations were estimated using BAYESASS (Wilson & Rannala, 2003). The migration rates were calculated between the populations identified by STRUCTURE and DAPC, and then reestimated with the individual biopsied in the Shannon Estuary but genetically assigned to *Coastal mobile* population grouped together with the Shannon dolphins. The MCMC mixing parameters of migration rates, allele frequencies, and inbreeding coefficients, were adjusted as recommended by Rannala (2007), during preliminary runs to obtain acceptance rates of around 30%. Ten runs with a burn‐in of 1,000,000 iterations followed by 10,000,000 MCMC iterations sampling every 1,000 iterations were performed. Convergence and mixing of chains were confirmed by plotting trace files using TRACER (Rambaut et al., 2014), and the consistency of runs was checked.

### 2.6 Effective population size

An estimate of contemporary effective population size (*N*_*e*_) for the *Coastal Shannon* population was derived using LDNe, a method that uses linkage disequilibrium (Waples & Do, 2008). This method has performed best in situations with little to no migration (<1%) (Gilbert & Whitlock, 2015) and adequately with migration rates of up to ~5%–10% (Waples & England, 2011). Allele frequencies of <0.02 were excluded from the analyses to avoid bias caused by rare alleles (Louis, Viricel et al., 2014; Waples & Do, 2010). As some of the samples were collected over a 15‐year time period (in the Shannon Estuary) and the data are thus likely to be biased downward due to overlapping generations (Waples, 2010), the estimate of *N*_*e*_ was inflated by 15% as in Louis, Viricel et al. (2014). *N*_*e*_ could not be calculated for the *Coastal mobile* or the *Pelagic* populations, due to small sample size (Tallmon et al., 2010).

### 2.7 Analyses of social structure and site fidelity

To test possible drivers of population structure and connectivity, indices of social structure, site fidelity, and kinship were examined among the coastal bottlenose dolphins (*Shannon* and *Mobile*). Long‐term photo‐identification data are not available for the “pelagic” dolphins in this area. Social structure analyses were performed in SOCPROG 2.4 compiled version (Whitehead, 2009). The dataset was limited to photographs of sufficient quality (grades 1–3) and to individuals with permanent and obvious markings (mark severity grade M1, Figure 3) in order to identify individuals between several years, and only dolphins photographed in at least five separate encounters were included to reduce bias caused by rarely seen individuals (Whitehead, 2008). Individuals photographed together during an encounter were considered associated with each other, so an encounter was chosen as the grouping variable in SOCPROG. “Day” was chosen as the sampling period.

The strength of association between pairs of individuals (i.e., dyads) was measured using two indices of the frequency of co‐occurrence: the half‐weight association index (HWI) and the simple ratio (Cairns & Schwager, 1987; Ginsberg & Young, 1992). The simple ratio index is suitable when association is defined by the presence in the same group during a sampling period (Ginsberg & Young, 1992). However, the HWI can be more appropriate when not all individuals within a group have been identified (Ginsberg & Young, 1992), as is often the case with dolphin photo‐identification studies due to individuals reacting differently to the presence of the research vessel. As both indices gave almost identical results and were considered good representations of social structure by the high cophenetic correlation coefficient (CCC) values (CCC HWI: 0.874, CCC simple ratio: 0.887), only the results derived using the HWI are presented. NETDRAW (Borgatti, 2002) was used to visualize a social network diagram using the network statistics calculated in SOCPROG. Permutation tests (Bejder, Fletcher, & Bräger, 1998; Whitehead, 1999) with 20,000 steps were used to test whether the observed association patterns were different than expected from random associations and to identify dyads with significantly larger or smaller association indices.

The standardized lagged association rate (SLAR) was used to test if temporary or long‐lasting social bonds existed between individuals, and compared to the null association rate (expected if all individuals are associating at random). The SLAR was fitted separately to the individuals encountered within and outside of the Shannon Estuary as the data showed that these groups did not associate with each other. Mathematical models representing simulated social structures, that is whether individuals had constant companionships or casual associates during the study (Whitehead, 1995), were fitted to the SLARs. The best fitting models were chosen based on the lowest quasi‐Akaike information criterion (QAIC) value (see Whitehead, 2007). To investigate movements of dolphins between different coastal areas and to estimate the amount of time identified individuals resided within each area, Lagged identification rates (LIRs) within and between all study areas were calculated in SOCPROG (Whitehead, 2009). Markov movement models (expected LIRs) of emigration/mortality and emigration + reimmigration (Whitehead, 2001) were fitted to estimate the probabilities of individuals moving from one area to another, and QAIC values were used to identify the best fitting model. 100 bootstrap replicates were used to estimate the standard error for the LIRs.

### 2.8 Relatedness, associations, and spatial overlap

A Mantel test in R package ade4 (Dray & Dufour, 2007) was used to investigate whether associations reflected kinship bonds, and whether a correlation existed between the strength of pairwise association (HWI) and relatedness between all biopsied dyads that had been encountered at least three times. To examine whether there was a correlation between spatial overlap and relatedness kernel utilization distribution (KUD) was calculated for individually identified dolphins that were encountered at least five times using R package adehabitatHR (Calenge, 2006), and the overlap in the areas used by two dolphins was then estimated by calculating the volume of intersection (VI) index (Fieberg & O'Kochanny, 2005; Podgórski, Lusseau, Scandura, Sönnichsen, & Jędrzejewska, 2014) of KUD. This index takes values between 0 and 1, and it quantifies the similarity between two KUDs thus comparing the area shared and the intensity of use by two individuals. These correlation tests were performed for the combined dataset and also separately for each of the two coastal populations, and significance tested in the correlations by performing randomization tests with 10,000 MCMC permutations.

## 3 RESULTS

Twelve new individuals, including ten coastal biopsies and two stranded dolphins, were genotyped for this study and analyzed together with 85 previously genotyped unique individuals from Mirimin et al. (2011). The dataset consisted of 32 females, 64 males, and one individual for which the sex could not be determined. Genotyping was successful in over 96% of cases with just 54 genotypes missing from the entire dataset of 1455. The probability (PI) of two of the 97 individuals sharing the same genotype over the 15 microsatellite loci was 4.5 × 10^−14^ for any two random unrelated individuals and 5.9 × 10^−6^ for siblings. This indicates that the set of markers used in this study has a high power to discriminate between identical genotypes that may have originated by chance alone. No identical genotypes were found among the samples genotyped in this study. When all the samples were pooled and tested for deviations from HWE across all microsatellite loci, eleven of the fifteen loci were found to be out of HWE. Further tests using MICRODROP (Wang et al., 2012) indicated no correlation between the amount of homozygotes and the amount of missing data across individuals (Pearson *r *=* *−0.091, *p *=* *0.85) or across loci (Pearson *r *=* *0.178, *p *=* *0.26), suggesting that homozygosity was not due to allelic dropout. Therefore, the observed deviations from HWE across all populations and loci are most likely attributed to the structuring of the populations, i.e., Wahlund effect (Wahlund, 1928). When deviations from HWE were inspected for each population separately, only two loci (*Dde*66 and *Dde*72) within the *Coastal mobile* population and one locus (*Dde*61) within the *Pelagic* population were out of HWE (Supporting information Appendix S2). STRUCTURE was therefore run with and without these three loci.

### 3.1 Individual assignment tests

**Figure 4:**
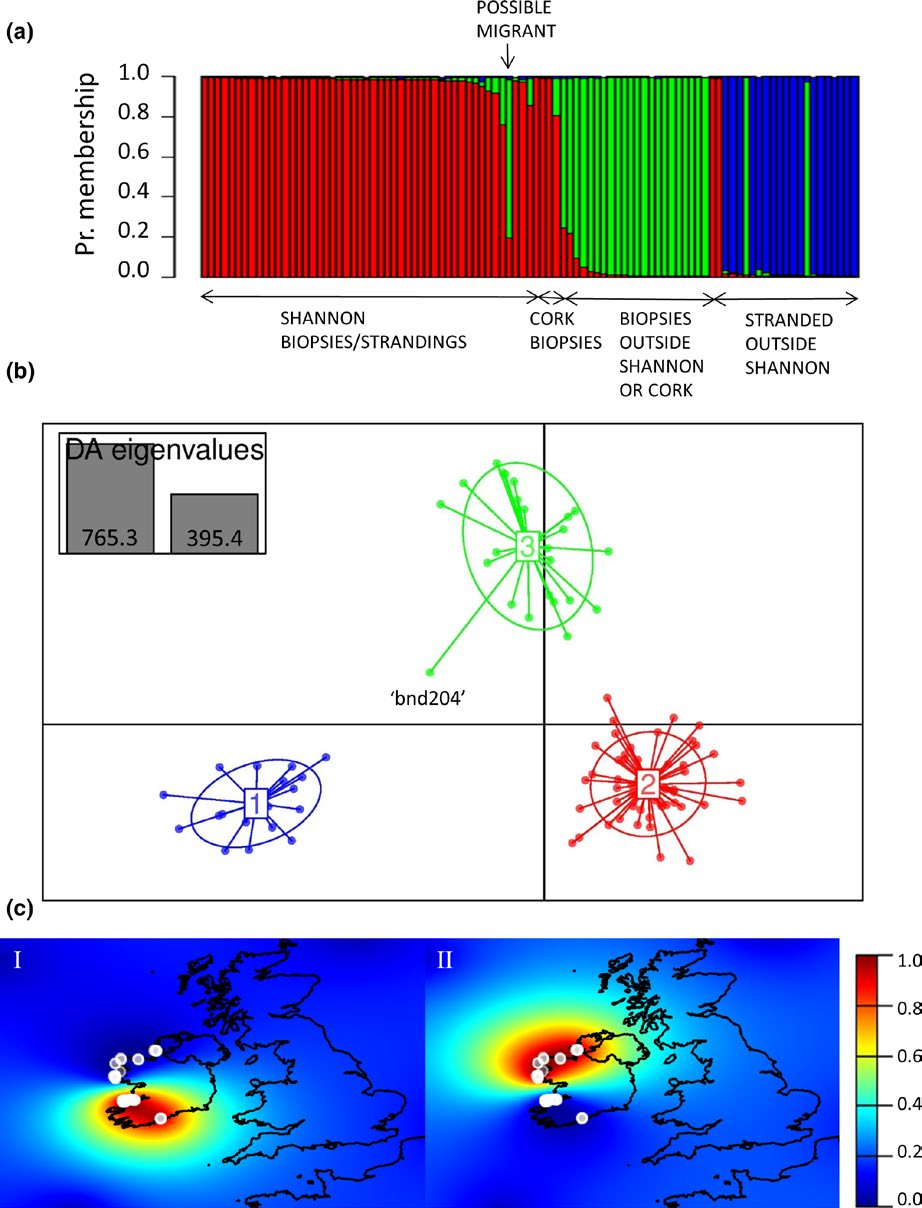
(a) Genetic assignment probabilities from STRUCTURE (*n *=* *97) with each vertical column corresponding to an individual dolphin and the colors indicating the membership proportions to each of the three clusters. (b) DAPC scatterplot clustering the samples (*n *=* *97) according to their first two principal components. The outlier “bnd204” was the only sample assigned differently by DAPC and STRUCTURE. Red, green, and blue colors represent *Coastal Shannon*,* Coastal Mobile,* and *Pelagic* dolphins, respectively. (c) Map of individual assignment probabilities per population, (I) *Coastal Shannon* and (II) *Coastal mobile* identified by TESS including only coastal biopsies (*n *=* *71). The color scale bar indicates the assignment probabilities. The results are based on analyses run with the complete set of 15 microsatellite loci

The most likely number of clusters (i.e., populations), *K*, identified by STRUCTURE based on the highest Pr(*X|K*) and using the ad hoc method by Evanno et al. (2005) was three when all the coastal biopsies and stranded samples were included in the analysis (Supporting information Appendix S3a). The majority of the individuals (92 of 97) were strongly assigned (with probability >90%) to one of these three clusters (Figure 4a). Removing the three loci that were out of HWE did not have an effect on the most likely number of clusters or the assignment of individuals into the three clusters. However, when considering assignments at *K *=* *2, the *Coastal mobile* dolphins clustered together with the *Pelagic* dolphins with high (>80%–90%) assignment probabilities instead of clustering together with the *Coastal Shannon* as was the case when all loci were included (latter presented in Supporting information Appendix S4a). This may have resulted from the large number of unique alleles only found in the pelagic samples (altogether 13 unique alleles) being left out of the analysis.

**Figure 5:**
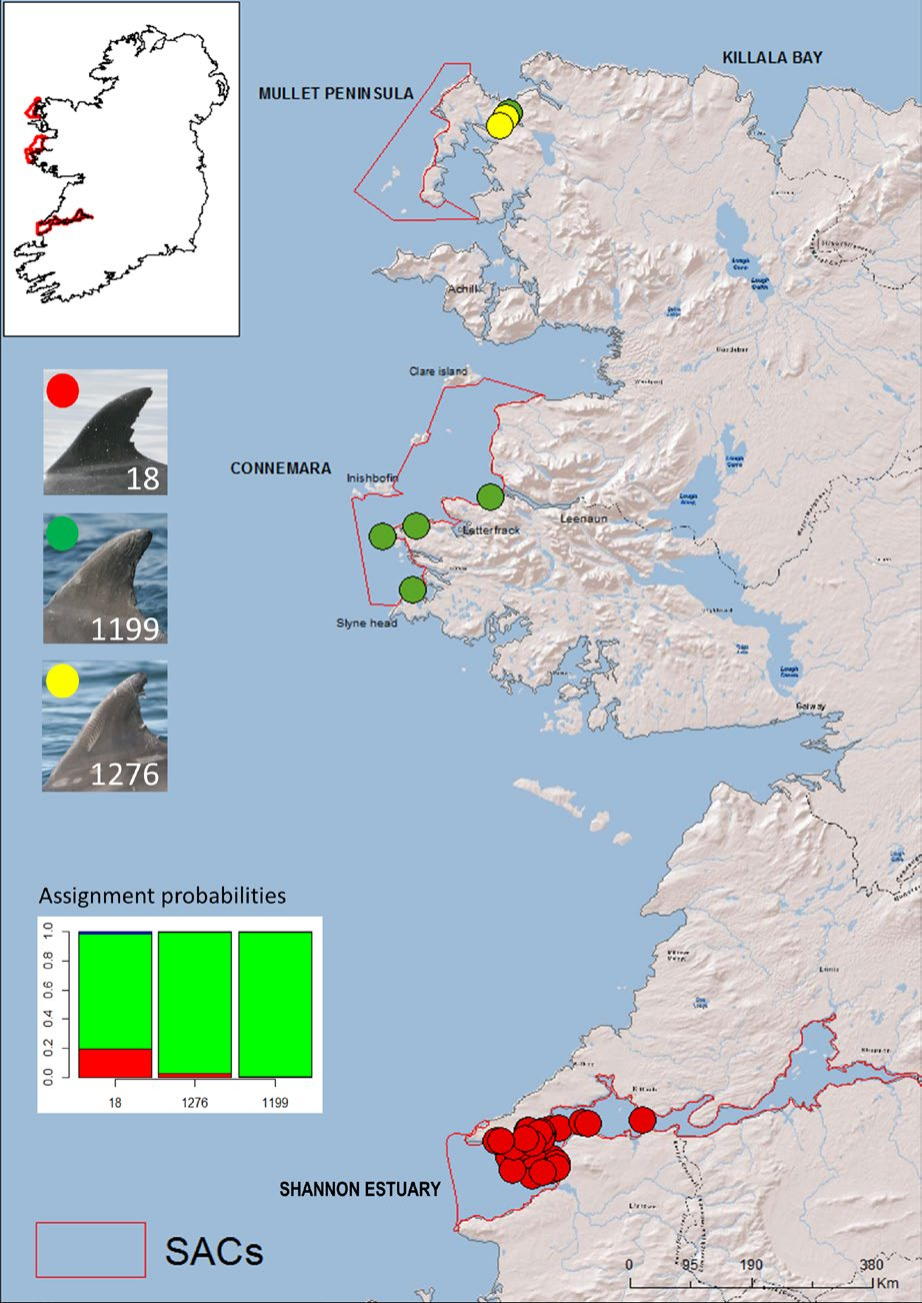
Possible migrant dolphin (a male given photo‐identification number #18) has been encountered only within Shannon Estuary SAC over 9 years (encounter locations indicated with red dots) but is genetically assigned to coastal mobile population with 79% certainty (green color in assignment probability plot from STRUCTURE). Dolphin #1276 (encounter locations indicated with green dots) is a male potentially closely related to #18 (*r *≥* *0.45), and he in turn is closely related to #1199 (encounter locations indicated with yellow dots), also a male. Both #1276 and #1199 are strongly assigned to the coastal mobile population

One individual (DNA sample code “tt‐05‐03” and photo‐identification number 18, see Figure 5) biopsy sampled inside the Shannon Estuary was assigned to the *Coastal mobile* cluster with 79% probability by STRUCTURE (individual indicated in Figure 4a, and in Supporting information Appendix S5, as a possible migrant; this was also found by Mirimin et al. (2011)). Four dolphins sampled in Cork Harbour were strongly assigned (>80% probability) to the same cluster as the *Coastal Shannon* dolphins (Figure 4a and Supporting information Appendix S5), consistent with Mirimin et al. (2011). Two individuals found dead‐stranded outside of the Shannon Estuary (~30 km and ~50 km north of the mouth of the estuary) were assigned to the *Coastal Shannon* population (Figure 4a); this may be a result of carcass drifting or an indication that at the least some of the *Coastal Shannon* population are using areas beyond the estuary.

DAPC, which does not assume HWE, also identified three clusters when all the samples were included (Supporting information Appendix S6) with a mild hierarchical structure among them; the distance between the clusters of *Coastal Shannon* and *Coastal mobile* samples is shorter than the distance between either of the coastal clusters and the *Pelagic* cluster (Figure 4b). Individual assignments were high (>99%) and highly consistent compared to STRUCTURE with 99% of the individuals assigned to the same cluster across the methods. In fact, only one stranded individual (sample code “bnd204,” an outlier in Figure 4b) was assigned to the *Coastal mobile* cluster by DAPC whereas it was clustered together with stranded pelagic samples by STRUCTURE when all the samples were included (Figure 4a).

These results were consistent with clustering probabilities calculated in TESS when only the biopsy samples of coastal dolphins (*n *=* *71) were considered; the most likely number of coastal populations identified was two (Figure 4c) as indicated by the DIC values reaching a plateau (Supporting information Appendix S7). The individual assignment probabilities were also 100% consistent with STRUCTURE and DAPC with all the same individuals assigned with >90% probability to either the *Coastal Shannon* or the *Coastal mobile* cluster (excluding the individual sampled in the Shannon Estuary that assigned to the *Coastal mobile* cluster with 59% certainty).

The samples assigned to the *Coastal Shannon* population had the largest percentage (2.4%) of dyads that were close relatives, with the Queller and Goodnight (1989) relatedness coefficient *r *≥* *0.45 indicating possible parent–offspring or full sibling relationships among these individuals. Relatedness was also found in the *Coastal mobile* cluster, with 2.0% of all possible dyads assigned as being close relatives; no close relatives were found among the *pelagic* samples. The mean relatedness coefficient varied from −0.02 (*SD* = 0.23) among individuals assigned to the *Coastal Shannon* population, −0.04 (*SD* = 0.25) among the *Coastal mobile*, to −0.06 (*SD* = 0.13) among the *Pelagic* dolphins. The mean relatedness values within the *Coastal Shannon* (1,431 possible dyads) and the *Coastal mobile* (300 dyads) were also significantly higher compared to the relatedness of dyads when individuals were selected randomly, one from each of the two coastal populations (1,350 dyads, Kruskal–Wallis *p *<* *0.0001, Supporting information Appendix S8).

Removing one individual from a dyad with relatedness coefficient *r *≥* *0.45 led to the removal of 22 individuals from the *Coastal Shannon* and six individuals from the *Coastal mobile* cluster. When considering only these “coastal” samples, the most likely number of clusters identified by STRUCTURE and the Evanno method was still two (Supporting information Appendix S3b,d) and the majority of individuals (49 of 51) were assigned to either of the two coastal populations with >80% certainty (Supporting information Appendix S4b). However, when including samples from all three populations after removing close relatives, the most likely number of populations was two with a division of samples to coastal and pelagic clusters (Supporting information Appendices S3c and S4b), indicating that relatedness may be a significant driver of finer scale population structuring.

### 3.2 Population differentiation and effective population size

No evidence of significant heterozygote deficiency was found across all loci in any of the populations (*Coastal Shannon p *=* *0.998, *Pelagic p *=* *0.469, *Coastal mobile p *=* *0.061). Allele richness (AR) and observed heterozygosity (*H*_*O*_) were lower in the two *coastal* populations compared to the *pelagic* population (Supporting information Appendix S2). Inbreeding coefficients were low in all populations. The mean estimate for effective population size in the *Coastal Shannon* population was 32 (with 95% CI of 22–43).

There was significant differentiation in allele frequencies (based on both *F*_ST_ and Jost's *D*) between the *pelagic* and the two *coastal* populations and between the two coastal populations (defined with STRUCTURE), and this difference persisted after removing close relatives from the dataset (Table 1). The Jost's *D* values revealed a hierarchical population structure, with largest differences observed between the *pelagic* and the two *coastal* populations (Table 1). The pairwise comparisons of *F*_ST_ values for randomized *coastal* populations showed no population differentiation when two sets of 10 individuals were randomly drawn from within the same population, *that is* consisting of only *Coastal Shannon* (mean: −0.0005, 95% CI: −0.0086–0.0080) or *Coastal mobile* (mean: 0.0021, 95% CI: −0.0074–0.0115) individuals (Supporting information Appendix S9). However, significant population differentiation was observed in comparisons of 10 individuals randomly drawn from one population with 10 individuals randomly drawn from the other (mean *F*_ST_: 0.1820, 95% CI: 0.1589–0.2051) indicating a true population differentiation that was not driven by the sampling of closely related individuals or uneven sampling. The simulations run in POWSIM 4.1 (Ryman & Palm, 2006) indicated that the power to detect a differentiation of *F*_ST_ ≥ 0.1 between the two coastal populations was >0.99 with the set of 15 microsatellite markers used in the present study, even with a low sample size of 10 individuals drawn from each population.

**Table 1:** Pairwise *F*_ST_ values based on 15 microsatellite loci (given as average with 95% HPDI) between the different populations *Coastal Shannon*,* Coastal mobile,* and *Pelagic*

	*Coastal Shannon*	*Pelagic*	*Coastal mobile*
*F*_ST_			
* Coastal Shannon*	–	0.173 (0.151–0.200)	0.181 (0.147–0.218)
* Pelagic*	0.154 (0.131–0.181)	–	0.186 (0.154–0.222)
* Coastal mobile*	0.161 (0.121–0.205)	0.172 (0.139–0.209)	–
Jost's *D*			
* Coastal Shannon*	–	0.362 (0.304–0.426)	0.207 (0.165–0.251)
* Pelagic*	0.339 (0.279–0.404)	–	0.319 (0.265–0.378)
* Coastal mobile*	0.188 (0.137–0.244)	0.305 (0.250–0.369)	–

Notes

The samples were divided into populations based on results from STRUCTURE. Values above the diagonal are for the whole dataset, and values below the diagonal are after removal of close relatives (*r *≥* *0.45).

### 3.3 Sex‐biased dispersal and migration rates

No evidence of sex‐biased dispersal was found in any of the indices used (Supporting information Appendix S10). The inferred migration rates (the proportion of migrants per population) calculated with BAYESASS were nonsignificant as zero was included in the range of 95% confidence intervals in each comparison (Table 2).

**Table 2:** Inferred (posterior) mean migration rates (with 95% HPDI) between the different Irish bottlenose dolphin populations identified by STRUCTURE and DAPC, given as proportion of migrants per population

	Sink		
Source	*Coastal Shannon*	*Pelagic*	*Coastal mobile*
*Coastal Shannon*	0.987 (0.969–1.000)	0.006 (−0.005–0.017)	0.008 (−0.007–0.022)
*Pelagic*	0.016 (−0.014–0.046)	0.948 (0.892–1.000)	0.036 (−0.014–0.086)
*Coastal mobile*	0.034 (−0.011–0.078)	0.012 (−0.010–0.034)	0.955 (0.906–1.000)

Note

Values for self‐recruitment are given in diagonal.

When looking at individual posterior probabilities of migrant ancestry, two individuals from the *Coastal mobile* population and one from the *Pelagic* population had >50% probability of being either first‐ or second‐generation migrants from other populations. Two individuals from the *Coastal mobile* population (“tt‐09‐12” and “12‐09‐2014_Tt2”) were second‐generation migrants from the *Coastal Shannon* population with 64% and 79% probability, respectively. One individual assigned to the *Pelagic* population by STRUCTURE (“bnd204”) had a 37% probability of being a first‐generation migrant and a 46% probability of being a second‐generation migrant from the *Coastal mobile* population. When the individual that was biopsied in the Shannon Estuary but genetically assigned to *Coastal mobile* population (“tt‐05‐03”) was grouped together with other Shannon individuals, it had a 19% probability of being a first‐generation migrant and a 70% probability of being a second‐generation migrant from the *Coastal mobile* population.

### 3.4 Social structure and site fidelity

**Figure 6:**
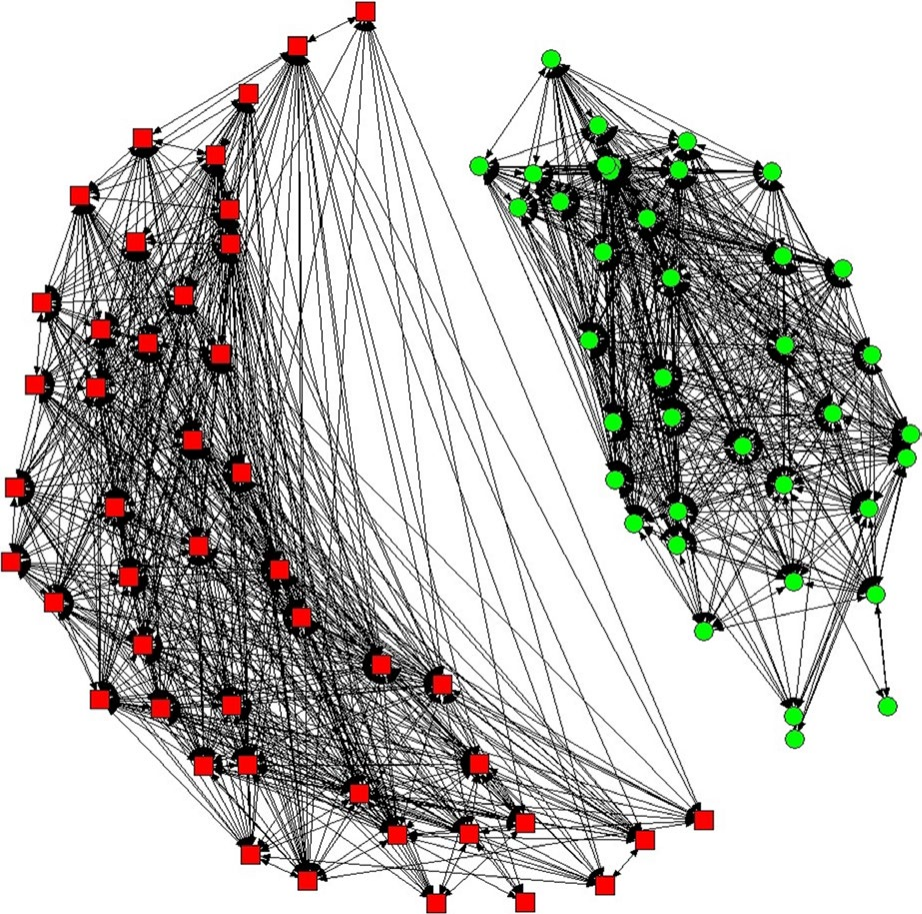
Social network diagram of bottlenose dolphins encountered on at least five occasions during the data collection 1996–2014. Boxes represent a social cluster of individuals encountered in the Shannon Estuary, and circles a cluster of the “mobile” dolphins encountered on the west and north‐west coast of Ireland. The length of the line in the network diagram inversely represents the strength of the association between a dyad calculated as half‐weight index (HWI)

When testing for preferred and avoided companionships between and within the two coastal populations, the mean HWI in the real data was found to be significantly higher compared to the HWI of a permuted random dataset (mean: *p *<* *0.01, *SD*:* p *<* *0.0001, and CV: *p *<* *0.0001) indicating significant preferred short‐ (within sampling period) and long‐term (between sampling periods) companions. Moreover, the proportion of nonzero elements was larger in the random data compared to real data which suggests that some individuals may avoid others (Whitehead, 2009), both within each population and between the two coastal populations (Figure 6). The latter comes as no surprise as the two populations have not been documented associating with each other. Pairwise associations within the *Coastal Shannon* population were best described by the standardized lagged association rate (SLAR) model “casual acquaintances” (Supporting information Appendix S11a), by which dyads remain associated for a period of time, dissociate and may, or may not, reassociate (Whitehead, 2015; Whitehead, Waters, & Lyrholm, 1991). Within the *Coastal mobile* population, on the other hand, the model “constant companions and casual acquaintances” best explained the data, with “constant companions” remaining associated with each other throughout the length of the study (Whitehead, 2015; Whitehead et al., 1991) (Supporting information Appendix S11b). The mean HWI within the *Coastal Shannon* was 0.08 (*SD* = 0.09) and within the *Coastal mobile* population it was 0.23 (*SD* = 0.21). The difference in the association indices between the two populations and especially the higher variation associated with the *Coastal mobile* may be linked to the lower number of encounters included in the social analysis (48 with the *Coastal mobile* and 315 with the *Coastal Shannon*).

**Figure 7:**
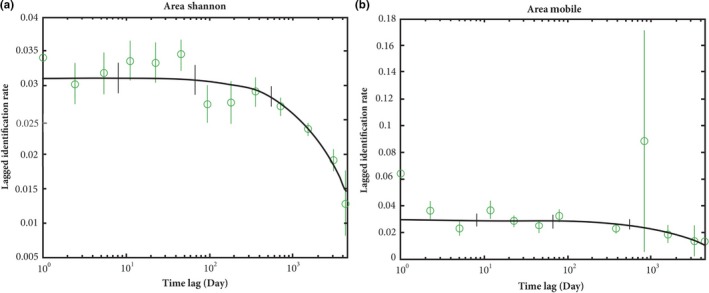
Lagged identification rate (LIR) for bottlenose dolphins encountered ≥5 times (a) in the Shannon Estuary and (b) outside the Shannon Estuary in the coastal waters of Ireland during the study period 1996–2014. The graph describes the probability that a dolphin photographed at time 0 will be identified again at time X within the area. Data points are represented as green circles (with SE), and the best fitting model (see Supporting information Appendix S12) is displayed as the black solid line. Time lag (number of days) is given on logarithmic scale

Bottlenose dolphins that were first photographed in the Shannon Estuary were not photographed anywhere else during 1996–2008 except once in Brandon Bay, Co. Kerry (approximately 15 km south from the mouth of the Shannon Estuary), hence their annual average lagged identification rate (LIR) was zero to any other study area, except to Brandon Bay where it was 0.0263 (*SE* = 0.0128). Likewise, dolphins belonging to the *Coastal mobile* population were never photographed in the Shannon Estuary during the study period so their LIR in the Shannon Estuary was also zero. The LIR within the Shannon stayed fairly constant for approximately 100 days, followed by some fluctuations in the rate (Figure 7a). Two competing models had substantial support explaining the data, with the emigration/mortality model having the lowest AIC value, followed by emigration + reimmigration + mortality model (Supporting information Appendix S12). LIR associated with the *Coastal mobile* population was best explained by the emigration/mortality model (Figure 7b, Supporting information Appendix S12).

### 3.5 Relatedness, spatial overlap, and associations

When only the biopsied individuals with a sufficient number of photo‐identification encounters (≥3) were considered, a significant correlation was found between the relatedness coefficient (Queller & Goodnight, 1989) and HWI (*r *=* *0.345, *p *=* *0.0001) when the data from the two coastal populations were combined. However, this is likely attributed to the correlation of zero values in the combined dataset as no correlation was found between the two indices when testing for this separately for each population (*Coastal Shannon: r *=* *0.028, *p *=* *0.363; *Coastal mobile: r *=* *0.0004, *p *=* *0.480). Of fifteen dyads with significant associations (i.e., who associated with each other significantly more or less than with other individuals), none had relatedness coefficient ≥0.45, but three dyads had coefficient values close to 0.25 indicating possible half‐siblings or cousins. No correlation was found between relatedness and spatial overlap within the *Coastal Shannon* (*r *=* *0.076, *p *=* *0.193) or the *Coastal mobile* population (*r *=* *0.042, *p *=* *0.417). Overall, these results indicate that close kinship may not strongly promote overall social associations in these two populations.

## 4 DISCUSSION

Understanding the scale of dispersal is an important consideration for the conservation and management of marine species (Lotterhos, 2012). By combining genetic and photo‐identification data, spatial dispersal and genetic dispersal over both short and long temporal scales have been elucidated in unprecedented detail for bottlenose dolphins in Irish waters. Dispersal can be gametic, *that is,* via gene flow during temporary interactions and spatial overlap, and therefore only detected by genetic methods. Dispersal can also be demographic, *that is,* the permanent movement of individuals from one location to another, detectable over the short‐term using photo‐identification of naturally marked individuals and over the past few generations using genetic methods (relatedness, migration, and admixture proportions; Iacchei et al., 2013). The combined results indicate social and reproductive isolation between the three identified populations, with only low levels of demographic and potential genetic connectivity sensu Lowe and Allendorf (2010). The accumulation of differentiation, estimated with fixation indices, indicates that this relative isolation has persisted over longer timescales.

Among the bottlenose dolphin samples, large and significant *F*_ST_ and Jost's *D* values between the populations, comparison of *F*_ST_ values from randomized “coastal populations,” the individual assignment methods, and kinship methods were all in agreement, supporting the division of the samples into one “*pelagic”* and two “*coastal”* clusters. In addition, Jost's *D* values and DAPC indicated the presence of a hierarchical population structure with the largest genetic difference occurring between the “*pelagic”* and “*coastal”* populations. Furthermore, social structure analyses using long‐term photo‐identification data revealed that the two coastal populations were not only genetically, but also socially, distinct. This kind of social separation has been previously reported between the “*pelagic”* and “*coastal”* bottlenose dolphins (Oudejans, Visser, Englund, Rogan, & Ingram, 2015).

The results also suggest that both coastal populations show a similar degree of site fidelity to their respective areas and are likely to have nonoverlapping core home ranges, at least during the seasons that photo‐identification work was conducted. The gradual decline in the lagged identification rates (LIRs) toward the end of the study period reflects a decrease in site fidelity that is likely explained by mortality and/or emigration. These results highlight that a high degree of site fidelity, especially evident in the Shannon Estuary SAC where data have been collected for over 12 years, is a key driver of fine‐scale population structure among coastal populations. A high degree of site fidelity among resident populations of bottlenose dolphins to certain local areas has been found in other parts of the world (Bristow & Rees, 2001; Möller, Allen, & Harcourt, 2002; Simoes‐Lopes & Fabian, 1999). This residency, found especially in embayments, coupled with genetic differentiation between dolphins residing in adjacent coastal habitats, has led a number of authors to suggest that variability in these habitats accompanied by the ability of local populations to accommodate it by the development of different foraging strategies (e.g., Barros & Wells, 1998; Smolker, Richards, Connor, Mann, & Berggren, 1997), may have shaped the fine‐scale population structure among these dolphins (Hoelzel et al., 1998; Chilvers & Corkeron, 2001; Natoli et al., 2005; Möller, Wiszniewski, Allen, & Beheregaray, 2007; Sargeant, Wirsing, Heithaus, & Mann, 2007; Richards et al., 2013; Allen et al., 2016). In addition, there is growing evidence that cultural transmission occurs within dolphin social communities in the form of social learning (e.g., Krützen et al., 2005; Mann, Stanton, Patterson, Bienenstock, & Singh, 2012) which may facilitate the evolution of specialist foraging behaviors, which in turn has the potential to maintain population structure between adjacent communities.

In this study, there is evidence of significant companionships within the two coastal populations, and it is possible that social bonds promote and maintain the observed social and genetic separation of these populations. The observed companionships did not seem to be linked to relatedness, but close associates were found both among kin and nonkin individuals, similar to a recent study by Louis et al. (2018). In contrast, close associations were linked to relatedness among females in a population of Indo‐Pacific bottlenose dolphins (Möller, Beheregaray, Allen, & Harcourt, 2006), and support for relatedness in male groups has been documented in alliances of this genus (Krützen et al., 2003), as well as among short‐beaked common dolphins (*Dephinus delphis*) in southern Australia, with greater relatedness found between males within schools than between schools (Zanardo, Bilgmann, Parra, & Möller, 2016). It is unfortunate that there were insufficient combined photo‐identification and genetic data to fully investigate possible sex‐specific patterns in the relatedness and associations among the two coastal Irish populations, partly due to genetic sampling being biased toward males (especially in the *Coastal Shannon* population) and partly because of the fact that the biopsy sampled animals did not necessarily have enough photo‐identification encounters for further social analyses.

Lowe and Allendorf (2010) described genetic connectivity as the exchange of alleles through gene flow between populations, and demographic connectivity as the dispersal of individuals from one population to another thus contributing to underlying population demographic processes and parameters (e.g., survival, mortality, abundance). Gene flow maintains genetic variation in populations, enhancing adaptive potential to environmental variation (Yamamichi & Innan, 2012). Even small amounts of gene flow can prevent the accumulation of large genetic differences between populations of low effective size (Palumbi, 2003; Slatkin, 1987). Hastings (1993), on the other hand, suggested that populations become demographically isolated if the exchange between populations stays below 10%, *that is,* <10% of the population growth is contributed by migrants from other populations regardless of whether they contribute to the gene flow or not. Recent migration rates between the different Irish bottlenose dolphin populations were nonsignificant (i.e., zero) in all comparisons inferred using BAYESASS. However, one individual (“tt05‐03”) encountered over 9 years in the Shannon Estuary, was genetically assigned to the *Coastal mobile* population. It is interesting that this dolphin has never been photographed associating with the *Coastal mobile* population, but no close kin were found among the sampled individuals assigned to the *Coastal Shannon* population. Given that ~40% of the *Coastal Shannon* population have been biopsied (and genotyped) based on abundance estimates derived for this population varying between 114 and 140 (Berrow, 2012; Berrow, Holmes, & Kiely, 1996; Englund, Ingram, & Rogan, 2007; Englund et al., 2008; Ingram & Rogan, 2002, 2003), it is possible that this dolphin has not (yet) genetically contributed to dispersal of gametes into the *Coastal Shannon* population. In contrast, close kinship was found between “tt05‐03” and an individual sampled within the *Coastal mobile* population. Thus, “tt05‐03” appears to be an example of demographic dispersal from the *Coastal mobile* population to the *Coastal Shannon* population. Nonetheless, considering that this individual (one of 46 biopsied dolphins in the Shannon Estuary) represents <3% demographic dispersal between the coastal Irish populations, it seems unlikely that the contribution to the demographic processes are significant. However, this largely depends on the management targets set to the population in question and the power to detect changes in abundance, survival, or other demographic processes.

No evidence for sex‐biased dispersal was found in this study. However, the sampling was biased toward males (due to efforts to sample marked animals), with more than double the amount of samples compared to females; thus these results should be treated with caution. Both Mirimin et al. (2011) and Louis et al. (2014a) found two haplotypes that were shared between “coastal” and “pelagic” dolphins based on the mitochondrial control region, but the sequencing of the entire mitochondrial genome revealed no shared haplotypes between these two “ecotypes” suggesting limited female dispersal between coastal and pelagic populations (Moura et al., 2013; Nykänen, 2016). However, two mitogenome haplotypes were shared between the *Coastal Shannon* and *Coastal mobile* populations (Nykänen, 2016), suggesting either that some movement between these populations exists via female‐mediated gene flow, or that the shared haplotypes are a consequence of shared ancestry and recent divergence between the two populations.

Two individuals strongly assigned to the *Coastal mobile* population were identified as likely second‐generation migrants originating from the *Coastal Shannon* population. However, whilst individual assignment methods, such as STRUCTURE, are believed to perform well at identifying migrant individuals (Putman & Carbone, 2014), BAYEASS was found to be less reliable in calculating individual migrant probabilities (Faubet, Waples, & Gaggiotti, 2007); thus, these results should be interpreted with caution. Nevertheless, BAYEASS was found to perform well at estimating overall migration rates between populations over a few generations at migration rates up to 0.1 (Faubet et al., 2007). Whether these dispersal events further translated into gene flow is uncertain and warrants more sampling effort especially within the *Coastal mobile* population. To date, only ~12% of this population occurring in Irish waters has been sampled, based on a median multisite abundance estimate of 189 dolphins derived for a wide area extending to the west and north‐west coast of Ireland (Nykänen, 2016). Overall, despite some evidence for low levels of demographic dispersal, it appears that connectivity between populations is too low to prevent the buildup of genetic differentiation.

Nichols et al. (2007) and Louis et al. (2014a) suggested that coastal bottlenose dolphins in northern European waters may form a wider metapopulation (the “*Coastal North”* metapopulation, Louis et al., 2014a) consisting of interconnected local populations around the British Isles. However, these studies did not have samples from the *Coastal Shannon* population, which is, based on this study, both genetically and demographically isolated. Coupled with the relatively small effective population size, this makes *Coastal Shannon* especially vulnerable to any environmental or anthropogenic stressors. The *Coastal mobile* population occurring in Irish waters, on the other hand, may belong to this “*Coastal North”* metapopulation, and previous research has shown that at least some of these mobile coastal animals travel over distances at the scale of hundreds of kilometers (Cheney et al., 2013; Ingram et al., 2001, 2003; O'Brien et al., 2009; Robinson et al., 2012). If they do indeed comprise part of the “*Coastal North”* metapopulation extending beyond Irish waters, transnational cooperation, monitoring and management may be needed. Six individuals from the west coast of Ireland have been matched on an ad hoc basis to photo‐identification catalogues comprised of animals ranging in the coastal waters of Scotland (Robinson et al., 2012) but there is a need for a consistent collaborative effort to better integrate photo‐identification catalogues from different regions/countries (e.g., Ireland, Wales, Scotland, France, Cornwall). Such collaboration would provide better insights into demographic dispersal, ranging patterns and the abundance of this putative metapopulation. In addition, genetic dispersal within the metapopulation needs to be quantified through increased sampling effort over a larger area extending beyond country boundaries and using a common set of genetic markers that are comparable between laboratories.

The present study supports the delineation of the three populations occurring in Irish waters as separate management units based on the low genetic, social, and demographic dispersal between the populations, thus validating the current designation of separate SACs for the two coastal populations. The study also highlights the importance of distinguishing genetic and demographic connectivity so that gene flow can be differentiated from immigration that has no subsequent genetic contribution from the migrant to the local population. Even though the genetic connectivity between the different populations of bottlenose dolphins in this study was negligible and accompanied by moderate‐to‐strong genetic differentiation, quantification of migration rates and the degree of social connectivity have implications in the delineation of MUs, especially in cases where population structuring is less clear. With this information, the functioning of existing marine protected areas or networks can be better assessed and the need for designating new protected areas is evaluated.

## CONFLICT OF INTEREST

None declared.

## AUTHOR CONTRIBUTIONS

M.N., A.D.F., S.N.I., and E.R. conceptualized the work and the analyses. E.D. and M.N. performed laboratory work. M.N., L.M., and M.L analyzed the genetic data. M.N., M.O., A.E., and S.N.I. analyzed the photo‐identification data. M.N., S.N.I., E.R., A.D.F., M.O., and A.E. collected the genetic samples and photo‐identification data. M.N. wrote the manuscript. All authors approved the final manuscript.

## DATA ACCESSIBILITY

Analysis input files used for TESS, DAPC, STRUCTURE, diveRsity, and SOCPROG are deposited in Dryad (Supporting information Data S1–S4).
