# Supplementary material for: Quantifying dispersal between marine protected areas by a highly mobile species, the bottlenose dolphin, Tursiops truncatus
Source: Ecol Evol. 2018 Aug 23;8(18):9241–58. doi: 10.1002/ece3.4343 (PMC6194238; doi:10.1002/ece3.4343)
Supplement: Supplementary file 1 [file ECE3-8-9241-s001.docx]

**Table S1.** Bottlenose dolphin photo-ID surveys done by University College Cork research teams in 1996-2014, including survey dates, approximate locations, and number of dolphin groups (encounters) recorded during each survey.

| Date | | Survey location | Survey area | Number of encounters |
| --- | --- | --- | --- | --- |
| 13/06/1996 | Shannon Estuary | | Shannon Estuary | 0 |
| 20/06/1996 | Shannon Estuary | | Shannon Estuary | 0 |
| 16/07/1996 | Shannon Estuary | | Shannon Estuary | 4 |
| 03/08/1996 | Shannon Estuary | | Shannon Estuary | 4 |
| 13/08/1996 | Shannon Estuary | | Shannon Estuary | 8 |
| 15/08/1996 | Shannon Estuary | | Shannon Estuary | 2 |
| 04/09/1996 | Shannon Estuary | | Shannon Estuary | 7 |
| 08/09/1996 | Shannon Estuary | | Shannon Estuary | 7 |
| 09/10/1996 | Shannon Estuary | | Shannon Estuary | 1 |
| 14/11/1996 | Shannon Estuary | | Shannon Estuary | 0 |
| 11/12/1996 | Shannon Estuary | | Shannon Estuary | 3 |
| 21/01/1997 | Shannon Estuary | | Shannon Estuary | 1 |
| 21/03/1997 | Shannon Estuary | | Shannon Estuary | 1 |
| 09/04/1997 | Shannon Estuary | | Shannon Estuary | 2 |
| 11/04/1997 | Shannon Estuary | | Shannon Estuary | 2 |
| 14/04/1997 | Shannon Estuary | | Shannon Estuary | 0 |
| 02/05/1997 | Shannon Estuary | | Shannon Estuary | 1 |
| 17/05/1997 | Shannon Estuary | | Shannon Estuary | 3 |
| 27/05/1997 | Shannon Estuary | | Shannon Estuary | 5 |
| 16/06/1997 | Shannon Estuary | | Shannon Estuary | 7 |
| 23/06/1997 | Shannon Estuary | | Shannon Estuary | 3 |
| 04/07/1997 | Shannon Estuary | | Shannon Estuary | 3 |
| 05/07/1997 | Shannon Estuary | | Shannon Estuary | 4 |
| 08/07/1997 | Shannon Estuary | | Shannon Estuary | 5 |
| 09/07/1997 | Shannon Estuary | | Shannon Estuary | 1 |
| 18/07/1997 | Shannon Estuary | | Shannon Estuary | 9 |
| 21/07/1997 | Shannon Estuary | | Shannon Estuary | 1 |
| 22/07/1997 | Shannon Estuary | | Shannon Estuary | 6 |
| 18/08/1997 | Shannon Estuary | | Shannon Estuary | 2 |
| 22/08/1997 | Shannon Estuary | | Shannon Estuary | 2 |
| 23/08/1997 | Shannon Estuary | | Shannon Estuary | 6 |
| 24/08/1997 | Shannon Estuary | | Shannon Estuary | 7 |
| 10/09/1997 | Shannon Estuary | | Shannon Estuary | 1 |
| 11/09/1997 | Shannon Estuary | | Shannon Estuary | 3 |
| 25/10/1997 | Shannon Estuary | | Shannon Estuary | 2 |
| 26/11/1997 | Shannon Estuary | | Shannon Estuary | 1 |
| 29/01/1998 | Shannon Estuary | | Shannon Estuary | 2 |
| 03/02/1998 | Shannon Estuary | | Shannon Estuary | 0 |
| 22/03/1998 | Shannon Estuary | | Shannon Estuary | 3 |
| 14/05/1998 | Shannon Estuary | | Shannon Estuary | 3 |
| 17/05/1998 | Shannon Estuary | | Shannon Estuary | 2 |
| 01/08/1998 | Shannon Estuary | | Shannon Estuary | 2 |
| 18/08/1998 | Shannon Estuary | | Shannon Estuary | 2 |
| 22/08/1998 | Shannon Estuary | | Shannon Estuary | 3 |
| 27/08/1998 | Shannon Estuary | | Shannon Estuary | 3 |
| 28/08/1998 | Shannon Estuary | | Shannon Estuary | 6 |
| 29/08/1998 | Shannon Estuary | | Shannon Estuary | 3 |
| 24/06/1999 | Shannon Estuary | | Shannon Estuary | 6 |
| 09/07/1999 | Shannon Estuary | | Shannon Estuary | 3 |
| 05/09/2000 | Shannon Estuary | | Shannon Estuary | 1 |
| 17/02/2001 | Shannon Estuary | | Shannon Estuary | 2 |
| 08/07/2001 | Shannon Estuary | | Shannon Estuary | 2 |
| 09/07/2001 | Shannon Estuary | | Shannon Estuary | 1 |
| 15/07/2001 | Killary Fjord, Connemara, Co. Galway | | West Coast (outside Shannon Estuary) | 2 |
| 16/07/2001 | Killary Fjord, Connemara, Co. Galway | | West Coast (outside Shannon Estuary) | 0 |
| 22/07/2001 | Castlegregory, Co. Kerry | | West Coast (outside Shannon Estuary) | 0 |
| 26/07/2001 | Shannon Estuary | | Shannon Estuary | 4 |
| 27/07/2001 | Shannon Estuary | | Shannon Estuary | 1 |
| 28/07/2001 | Shannon Estuary | | Shannon Estuary | 6 |
| 31/07/2001 | Shannon Estuary | | Shannon Estuary | 1 |
| 03/08/2001 | Donegal Bay, Co. Donegal | | West Coast (outside Shannon Estuary) | 0 |
| 04/08/2001 | Donegal Bay, Co. Donegal | | West Coast (outside Shannon Estuary) | 0 |
| 05/08/2001 | Broadhaven Bay, Co. Mayo | | West Coast (outside Shannon Estuary) | 0 |
| 07/08/2001 | Broadhaven Bay, Co. Mayo | | West Coast (outside Shannon Estuary) | 0 |
| 09/08/2001 | Killary Fjord, Connemara, Co. Galway | | West Coast (outside Shannon Estuary) | 0 |
| 10/08/2001 | Donegal Bay, Co. Donegal | | West Coast (outside Shannon Estuary) | 1 |
| 17/08/2001 | Shannon Estuary | | Shannon Estuary | 5 |
| 06/09/2001 | Shannon Estuary | | Shannon Estuary | 1 |
| 10/09/2001 | Castlegregory, Co. Kerry | | West Coast (outside Shannon Estuary) | 3 |
| 11/09/2001 | Shannon Estuary | | Shannon Estuary | 0 |
| 20/09/2001 | Killary Fjord, Connemara, Co. Galway | | West Coast (outside Shannon Estuary) | 1 |
| 21/09/2001 | Broadhaven Bay, Co. Mayo | | West Coast (outside Shannon Estuary) | 0 |
| 22/09/2001 | Shannon Estuary | | Shannon Estuary | 4 |
| 28/10/2001 | Shannon Estuary | | Shannon Estuary | 2 |
| 03/11/2001 | Shannon Estuary | | Shannon Estuary | 3 |
| 19/12/2001 | Shannon Estuary | | Shannon Estuary | 2 |
| 15/02/2002 | Shannon Estuary | | Shannon Estuary | 1 |
| 29/03/2002 | Shannon Estuary | | Shannon Estuary | 2 |
| 16/07/2002 | Shannon Estuary | | Shannon Estuary | 3 |
| 17/07/2002 | Shannon Estuary | | Shannon Estuary | 4 |
| 05/08/2002 | Shannon Estuary | | Shannon Estuary | 2 |
| 22/08/2002 | Shannon Estuary | | Shannon Estuary | 3 |
| 27/08/2002 | West Cork, Co. Cork | | West Coast (outside Shannon Estuary) | 0 |
| 29/08/2002 | Youghal, Co. Cork | | West Coast (outside Shannon Estuary) | 1 |
| 04/09/2002 | Shannon Estuary | | Shannon Estuary | 2 |
| 16/09/2002 | Shannon Estuary | | Shannon Estuary | 1 |
| 19/09/2002 | Connemara, Co. Galway | | West Coast (outside Shannon Estuary) | 1 |
| 20/09/2002 | Connemara, Co. Galway | | West Coast (outside Shannon Estuary) | 2 |
| 21/09/2002 | Connemara, Co. Galway | | West Coast (outside Shannon Estuary) | 1 |
| 25/09/2002 | West Cork, Co. Cork | | West Coast (outside Shannon Estuary) | 0 |
| 05/07/2003 | Shannon Estuary | | Shannon Estuary | 9 |
| 04/08/2003 | Shannon Estuary | | Shannon Estuary | 3 |
| 05/08/2003 | Shannon Estuary | | Shannon Estuary | 11 |
| 06/08/2003 | Shannon Estuary | | Shannon Estuary | 2 |
| 07/08/2003 | Shannon Estuary | | Shannon Estuary | 4 |
| 08/08/2003 | Shannon Estuary | | Shannon Estuary | 1 |
| 10/08/2003 | Shannon Estuary | | Shannon Estuary | 2 |
| 11/08/2003 | Shannon Estuary | | Shannon Estuary | 6 |
| 12/08/2003 | Shannon Estuary | | Shannon Estuary | 3 |
| 13/08/2003 | Shannon Estuary | | Shannon Estuary | 4 |
| 15/08/2003 | Shannon Estuary | | Shannon Estuary | 4 |
| 16/08/2003 | Shannon Estuary | | Shannon Estuary | 3 |
| 19/08/2003 | Cork Harbour, Co. Cork | | West Coast (outside Shannon Estuary) | 1 |
| 20/08/2003 | Cork Harbour, Co. Cork | | West Coast (outside Shannon Estuary) | 1 |
| 03/09/2003 | Killary Fjord, Connemara, Co. Galway | | West Coast (outside Shannon Estuary) | 0 |
| 04/09/2003 | Killary Fjord, Connemara, Co. Galway | | West Coast (outside Shannon Estuary) | 0 |
| 16/09/2003 | Shannon Estuary | | Shannon Estuary | 3 |
| 21/09/2003 | Shannon Estuary | | Shannon Estuary | 3 |
| 27/09/2003 | Connemara, Co. Galway | | West Coast (outside Shannon Estuary) | 1 |
| 28/09/2003 | Connemara, Co. Galway | | West Coast (outside Shannon Estuary) | 0 |
| 31/07/2004 | Kenmare River, Co. Kerry | | West Coast (outside Shannon Estuary) | 1 |
| 27/08/2005 | Shannon Estuary | | Shannon Estuary | 3 |
| 30/08/2005 | Shannon Estuary | | Shannon Estuary | 5 |
| 31/08/2005 | Shannon Estuary | | Shannon Estuary | 2 |
| 02/09/2005 | Cork Harbour, Co. Cork | | West Coast (outside Shannon Estuary) | 1 |
| 03/09/2005 | Cork Harbour, Co. Cork | | West Coast (outside Shannon Estuary) | 0 |
| 05/09/2005 | Shannon Estuary | | Shannon Estuary | 1 |
| 06/09/2005 | Shannon Estuary | | Shannon Estuary | 4 |
| 08/09/2005 | Shannon Estuary | | Shannon Estuary | 2 |
| 10/09/2005 | Shannon Estuary | | Shannon Estuary | 1 |
| 11/09/2005 | Shannon Estuary | | Shannon Estuary | 4 |
| 12/09/2005 | Shannon Estuary | | Shannon Estuary | 3 |
| 14/09/2005 | Shannon Estuary | | Shannon Estuary | 1 |
| 17/09/2005 | Shannon Estuary | | Shannon Estuary | 5 |
| 20/09/2005 | Shannon Estuary | | Shannon Estuary | 2 |
| 26/06/2006 | Shannon Estuary | | Shannon Estuary | 3 |
| 15/07/2006 | Shannon Estuary | | Shannon Estuary | 4 |
| 17/07/2006 | Shannon Estuary | | Shannon Estuary | 3 |
| 26/07/2006 | Shannon Estuary | | Shannon Estuary | 0 |
| 07/08/2006 | Shannon Estuary | | Shannon Estuary | 6 |
| 21/08/2006 | Shannon Estuary | | Shannon Estuary | 2 |
| 12/09/2006 | Shannon Estuary | | Shannon Estuary | 4 |
| 17/09/2006 | Shannon Estuary | | Shannon Estuary | 1 |
| 25/09/2006 | Shannon Estuary | | Shannon Estuary | 1 |
| 03/10/2006 | Shannon Estuary | | Shannon Estuary | 2 |
| 04/10/2006 | Shannon Estuary | | Shannon Estuary | 1 |
| 20/10/2006 | Shannon Estuary | | Shannon Estuary | 4 |
| 23/10/2006 | Shannon Estuary | | Shannon Estuary | 3 |
| 29/10/2006 | Shannon Estuary | | Shannon Estuary | 1 |
| 01/11/2006 | Shannon Estuary | | Shannon Estuary | 1 |
| 03/11/2006 | Shannon Estuary | | Shannon Estuary | 4 |
| 04/11/2006 | Shannon Estuary | | Shannon Estuary | 3 |
| 23/01/2007 | Shannon Estuary | | Shannon Estuary | 2 |
| 25/01/2007 | Shannon Estuary | | Shannon Estuary | 3 |
| 26/01/2007 | Shannon Estuary | | Shannon Estuary | 0 |
| 05/02/2007 | Shannon Estuary | | Shannon Estuary | 2 |
| 17/02/2007 | Shannon Estuary | | Shannon Estuary | 5 |
| 22/03/2007 | Shannon Estuary | | Shannon Estuary | 2 |
| 26/03/2007 | Shannon Estuary | | Shannon Estuary | 0 |
| 27/03/2007 | Shannon Estuary | | Shannon Estuary | 1 |
| 04/04/2007 | Shannon Estuary | | Shannon Estuary | 0 |
| 06/04/2007 | Shannon Estuary | | Shannon Estuary | 1 |
| 18/04/2007 | Shannon Estuary | | Shannon Estuary | 4 |
| 21/05/2007 | Cork Harbour, Co. Cork | | West Coast (outside Shannon Estuary) | 1 |
| 06/06/2007 | Shannon Estuary | | Shannon Estuary | 3 |
| 07/07/2007 | Shannon Estuary | | Shannon Estuary | 1 |
| 14/07/2007 | Shannon Estuary | | Shannon Estuary | 2 |
| 15/07/2007 | Shannon Estuary | | Shannon Estuary | 2 |
| 16/07/2007 | Shannon Estuary | | Shannon Estuary | 3 |
| 20/07/2007 | Shannon Estuary | | Shannon Estuary | 5 |
| 21/07/2007 | Shannon Estuary | | Shannon Estuary | 3 |
| 22/07/2007 | Shannon Estuary | | Shannon Estuary | 4 |
| 29/07/2007 | Shannon Estuary | | Shannon Estuary | 3 |
| 30/07/2007 | Shannon Estuary | | Shannon Estuary | 6 |
| 31/07/2007 | Shannon Estuary | | Shannon Estuary | 3 |
| 08/08/2007 | Shannon Estuary | | Shannon Estuary | 3 |
| 13/08/2007 | Shannon Estuary | | Shannon Estuary | 1 |
| 22/08/2007 | Shannon Estuary | | Shannon Estuary | 2 |
| 23/08/2007 | Shannon Estuary | | Shannon Estuary | 2 |
| 26/08/2007 | Shannon Estuary | | Shannon Estuary | 1 |
| 27/08/2007 | Shannon Estuary | | Shannon Estuary | 1 |
| 28/08/2007 | Shannon Estuary | | Shannon Estuary | 2 |
| 03/09/2007 | Shannon Estuary | | Shannon Estuary | 2 |
| 04/09/2007 | Shannon Estuary | | Shannon Estuary | 1 |
| 05/09/2007 | Shannon Estuary | | Shannon Estuary | 2 |
| 06/09/2007 | Shannon Estuary | | Shannon Estuary | 1 |
| 07/09/2007 | Shannon Estuary | | Shannon Estuary | 2 |
| 08/09/2007 | Shannon Estuary | | Shannon Estuary | 1 |
| 11/09/2007 | Shannon Estuary | | Shannon Estuary | 5 |
| 12/09/2007 | Shannon Estuary | | Shannon Estuary | 1 |
| 13/09/2007 | Shannon Estuary | | Shannon Estuary | 1 |
| 13/06/2008 | Cork Harbour, Co. Cork | | West Coast (outside Shannon Estuary) | 1 |
| 14/06/2008 | Shannon Estuary | | Shannon Estuary | 3 |
| 15/06/2008 | Dunmannus Bay, Co. Cork | | West Coast (outside Shannon Estuary) | 0 |
| 20/06/2008 | Cork Harbour, Co. Cork | | West Coast (outside Shannon Estuary) | 1 |
| 21/07/2008 | Cork Harbour, Co. Cork | | West Coast (outside Shannon Estuary) | 1 |
| 22/07/2008 | Cork Harbour, Co. Cork | | West Coast (outside Shannon Estuary) | 1 |
| 26/07/2008 | Valentia Island, Co. Kerry | | West Coast (outside Shannon Estuary) | 0 |
| 04/08/2008 | Shannon Estuary | | Shannon Estuary | 3 |
| 08/08/2008 | Shannon Estuary | | Shannon Estuary | 2 |
| 28/08/2008 | Cork Harbour, Co. Cork | | West Coast (outside Shannon Estuary) | 1 |
| 30/08/2008 | Shannon Estuary | | Shannon Estuary | 2 |
| 31/08/2008 | Shannon Estuary | | Shannon Estuary | 3 |
| 07/09/2008 | Shannon Estuary | | Shannon Estuary | 1 |
| 13/09/2008 | Shannon Estuary | | Shannon Estuary | 1 |
| 17/09/2008 | Shannon Estuary | | Shannon Estuary | 4 |
| 19/09/2008 | Connemara, Co. Galway | | West Coast (outside Shannon Estuary) | 0 |
| 20/09/2008 | Shannon Estuary | | Shannon Estuary | 0 |
| 23/09/2008 | Shannon Estuary | | Shannon Estuary | 0 |
| 24/09/2008 | Connemara, Co. Galway | | West Coast (outside Shannon Estuary) | 0 |
| 25/09/2008 | Connemara, Co. Galway | | West Coast (outside Shannon Estuary) | 0 |
| 26/09/2008 | Connemara, Co. Galway | | West Coast (outside Shannon Estuary) | 0 |
| 28/09/2008 | Shannon Estuary | | Shannon Estuary | 1 |
| 06/12/2008 | Cork Harbour, Co. Cork | | West Coast (outside Shannon Estuary) | 1 |
| 15/12/2008 | Dingle, Co. Kerry | | West Coast (outside Shannon Estuary) | 1 |
| 01/06/2009 | Connemara, Co. Galway | | West Coast (outside Shannon Estuary) | 1 |
| 02/06/2009 | Connemara, Co. Galway | | West Coast (outside Shannon Estuary) | 1 |
| 03/06/2009 | Connemara, Co. Galway | | West Coast (outside Shannon Estuary) | 1 |
| 04/06/2009 | Connemara, Co. Galway | | West Coast (outside Shannon Estuary) | 0 |
| 11/06/2009 | Cork Harbour, Co. Cork | | West Coast (outside Shannon Estuary) | 0 |
| 15/06/2009 | Cork Harbour, Co. Cork | | West Coast (outside Shannon Estuary) | 0 |
| 22/06/2009 | Connemara, Co. Galway | | West Coast (outside Shannon Estuary) | 2 |
| 23/06/2009 | Connemara, Co. Galway | | West Coast (outside Shannon Estuary) | 0 |
| 24/06/2009 | Connemara, Co. Galway | | West Coast (outside Shannon Estuary) | 1 |
| 29/06/2009 | Connemara, Co. Galway | | West Coast (outside Shannon Estuary) | 0 |
| 01/07/2009 | Mullet peninsula, Co. Mayo | | West Coast (outside Shannon Estuary) | 0 |
| 02/07/2009 | Mullet peninsula, Co. Mayo | | West Coast (outside Shannon Estuary) | 0 |
| 07/08/2009 | Connemara, Co. Galway | | West Coast (outside Shannon Estuary) | 0 |
| 12/08/2009 | Connemara, Co. Galway | | West Coast (outside Shannon Estuary) | 3 |
| 13/08/2009 | Connemara, Co. Galway | | West Coast (outside Shannon Estuary) | 0 |
| 10/09/2009 | Connemara, Co. Galway | | West Coast (outside Shannon Estuary) | 0 |
| 11/09/2009 | Connemara, Co. Galway | | West Coast (outside Shannon Estuary) | 1 |
| 12/09/2009 | Connemara, Co. Galway | | West Coast (outside Shannon Estuary) | 0 |
| 13/09/2009 | Mullet peninsula, Co. Mayo | | West Coast (outside Shannon Estuary) | 0 |
| 14/09/2009 | Mullet peninsula, Co. Mayo | | West Coast (outside Shannon Estuary) | 1 |
| 16/09/2009 | Connemara, Co. Galway | | West Coast (outside Shannon Estuary) | 0 |
| 17/09/2009 | Connemara, Co. Galway | | West Coast (outside Shannon Estuary) | 0 |
| 18/09/2009 | Connemara, Co. Galway | | West Coast (outside Shannon Estuary) | 1 |
| 11/03/2010 | Cork Harbour, Co. Cork | | West Coast (outside Shannon Estuary) | 1 |
| 03/05/2010 | Mullet peninsula, Co. Mayo | | West Coast (outside Shannon Estuary) | 0 |
| 26/06/2010 | Cork Harbour, Co. Cork | | West Coast (outside Shannon Estuary) | 0 |
| 28/07/2010 | Mullet peninsula, Co. Mayo | | West Coast (outside Shannon Estuary) | 1 |
| 29/07/2010 | Mullet peninsula, Co. Mayo | | West Coast (outside Shannon Estuary) | 0 |
| 07/09/2010 | Fanore, Co. Clare | | West Coast (outside Shannon Estuary) | 1 |
| 12/10/2010 | Mullet peninsula, Co. Mayo | | West Coast (outside Shannon Estuary) | 0 |
| 13/10/2010 | Mullet peninsula, Co. Mayo | | West Coast (outside Shannon Estuary) | 0 |
| 14/10/2010 | Mullet peninsula, Co. Mayo | | West Coast (outside Shannon Estuary) | 1 |
| 15/10/2010 | Mullet peninsula, Co. Mayo | | West Coast (outside Shannon Estuary) | 0 |
| 16/10/2010 | Cork Harbour, Co. Cork | | West Coast (outside Shannon Estuary) | 1 |
| 17/10/2010 | Cork Harbour, Co. Cork | | West Coast (outside Shannon Estuary) | 1 |
| 02/06/2011 | Cork Harbour, Co. Cork | | West Coast (outside Shannon Estuary) | 1 |
| 03/06/2011 | Cork Harbour, Co. Cork | | West Coast (outside Shannon Estuary) | 1 |
| 23/07/2011 | Connemara, Co. Galway | | West Coast (outside Shannon Estuary) | 1 |
| 24/07/2011 | Connemara, Co. Galway | | West Coast (outside Shannon Estuary) | 0 |
| 21/05/2013 | Connemara, Co. Galway | | West Coast (outside Shannon Estuary) | 1 |
| 24/05/2013 | Connemara, Co. Galway | | West Coast (outside Shannon Estuary) | 0 |
| 25/05/2013 | Connemara, Co. Galway | | West Coast (outside Shannon Estuary) | 1 |
| 29/05/2013 | Connemara, Co. Galway | | West Coast (outside Shannon Estuary) | 0 |
| 31/05/2013 | Connemara, Co. Galway | | West Coast (outside Shannon Estuary) | 0 |
| 02/06/2013 | Connemara, Co. Galway | | West Coast (outside Shannon Estuary) | 1 |
| 04/06/2013 | Connemara, Co. Galway | | West Coast (outside Shannon Estuary) | 1 |
| 06/06/2013 | Connemara, Co. Galway | | West Coast (outside Shannon Estuary) | 1 |
| 09/06/2013 | Donegal Bay, Co. Donegal | | West Coast (outside Shannon Estuary) | 0 |
| 12/06/2013 | Connemara, Co. Galway | | West Coast (outside Shannon Estuary) | 0 |
| 16/06/2013 | Connemara, Co. Galway | | West Coast (outside Shannon Estuary) | 0 |
| 19/06/2013 | Connemara, Co. Galway | | West Coast (outside Shannon Estuary) | 1 |
| 24/06/2013 | Connemara, Co. Galway | | West Coast (outside Shannon Estuary) | 0 |
| 25/06/2013 | Connemara, Co. Galway | | West Coast (outside Shannon Estuary) | 0 |
| 26/06/2013 | Connemara, Co. Galway | | West Coast (outside Shannon Estuary) | 1 |
| 05/07/2013 | Connemara, Co. Galway | | West Coast (outside Shannon Estuary) | 0 |
| 08/07/2013 | Mullet peninsula, Co. Mayo | | West Coast (outside Shannon Estuary) | 1 |
| 09/07/2013 | Mullet peninsula, Co. Mayo | | West Coast (outside Shannon Estuary) | 1 |
| 11/07/2013 | Mullet peninsula, Co. Mayo | | West Coast (outside Shannon Estuary) | 0 |
| 13/07/2013 | Connemara, Co. Galway | | West Coast (outside Shannon Estuary) | 1 |
| 14/07/2013 | Connemara, Co. Galway | | West Coast (outside Shannon Estuary) | 0 |
| 19/07/2013 | Connemara, Co. Galway | | West Coast (outside Shannon Estuary) | 0 |
| 20/07/2013 | Connemara, Co. Galway | | West Coast (outside Shannon Estuary) | 0 |
| 21/07/2013 | Connemara, Co. Galway | | West Coast (outside Shannon Estuary) | 0 |
| 25/07/2013 | Connemara, Co. Galway | | West Coast (outside Shannon Estuary) | 0 |
| 26/07/2013 | Connemara, Co. Galway | | West Coast (outside Shannon Estuary) | 0 |
| 05/08/2013 | Donegal Bay, Co. Donegal | | West Coast (outside Shannon Estuary) | 0 |
| 07/08/2013 | Donegal Bay, Co. Donegal | | West Coast (outside Shannon Estuary) | 1 |
| 08/08/2013 | Donegal Bay, Co. Donegal | | West Coast (outside Shannon Estuary) | 1 |
| 10/06/2014 | Connemara, Co. Galway | | West Coast (outside Shannon Estuary) | 0 |
| 12/06/2014 | Connemara, Co. Galway | | West Coast (outside Shannon Estuary) | 0 |
| 14/06/2014 | Connemara, Co. Galway | | West Coast (outside Shannon Estuary) | 0 |
| 17/06/2014 | Connemara, Co. Galway | | West Coast (outside Shannon Estuary) | 0 |
| 19/06/2014 | Connemara, Co. Galway | | West Coast (outside Shannon Estuary) | 1 |
| 20/06/2014 | Connemara, Co. Galway | | West Coast (outside Shannon Estuary) | 1 |
| 21/06/2014 | Connemara, Co. Galway | | West Coast (outside Shannon Estuary) | 1 |
| 24/06/2014 | Connemara, Co. Galway | | West Coast (outside Shannon Estuary) | 0 |
| 29/06/2014 | Connemara, Co. Galway | | West Coast (outside Shannon Estuary) | 1 |
| 30/06/2014 | Connemara, Co. Galway | | West Coast (outside Shannon Estuary) | 1 |
| 01/07/2014 | Connemara, Co. Galway | | West Coast (outside Shannon Estuary) | 1 |
| 10/07/2014 | Connemara, Co. Galway | | West Coast (outside Shannon Estuary) | 0 |
| 17/07/2014 | Connemara, Co. Galway | | West Coast (outside Shannon Estuary) | 0 |
| 20/07/2014 | Mullet peninsula, Co. Mayo | | West Coast (outside Shannon Estuary) | 0 |
| 23/07/2014 | Mullet peninsula, Co. Mayo | | West Coast (outside Shannon Estuary) | 0 |
| 24/07/2014 | Mullet peninsula, Co. Mayo | | West Coast (outside Shannon Estuary) | 0 |
| 27/07/2014 | Mullet peninsula, Co. Mayo | | West Coast (outside Shannon Estuary) | 1 |
| 01/08/2014 | Donegal Bay, Co. Donegal | | West Coast (outside Shannon Estuary) | 0 |
| 02/08/2014 | Donegal Bay, Co. Donegal | | West Coast (outside Shannon Estuary) | 0 |
| 04/08/2014 | Donegal Bay, Co. Donegal | | West Coast (outside Shannon Estuary) | 0 |
| 20/08/2014 | Donegal Bay, Co. Donegal | | West Coast (outside Shannon Estuary) | 1 |
| 22/08/2014 | Donegal Bay, Co. Donegal | | West Coast (outside Shannon Estuary) | 1 |
| 24/08/2014 | Donegal Bay, Co. Donegal | | West Coast (outside Shannon Estuary) | 1 |
| 26/08/2014 | Donegal Bay, Co. Donegal | | West Coast (outside Shannon Estuary) | 1 |
| 03/09/2014 | Mullet peninsula, Co. Mayo | | West Coast (outside Shannon Estuary) | 0 |
| 04/09/2014 | Mullet peninsula, Co. Mayo | | West Coast (outside Shannon Estuary) | 0 |
| 08/09/2014 | Killala Bay, Co. Mayo | | West Coast (outside Shannon Estuary) | 1 |
| 12/09/2014 | Donegal Bay, Co. Donegal | | West Coast (outside Shannon Estuary) | 1 |
| 13/09/2014 | Donegal Bay, Co. Donegal | | West Coast (outside Shannon Estuary) | 1 |
| 14/09/2014 | Donegal Bay, Co. Donegal | | West Coast (outside Shannon Estuary) | 0 |
