# Supplementary material for: Quantifying dispersal between marine protected areas by a highly mobile species, the bottlenose dolphin, Tursiops truncatus
Source: Ecol Evol. 2018 Aug 23;8(18):9241–58. doi: 10.1002/ece3.4343 (PMC6194238; doi:10.1002/ece3.4343)
Supplement: Supplementary file 2 [file ECE3-8-9241-s002.docx]

# Appendices

**Appendix 1.** The criteria used to score the quality of all photographs taken of dolphin dorsal fins (independent on degree of marking of individuals), from Englund et al. (2008).

| Grade | Criteria |
| --- | --- |
| 1 | Well lit and focused photo taken perpendicular to the dorsal fin at close range |
| 2 | More distant and less well lit or slightly angled photograph of the fin |
| 3 | Poorly lit or somewhat out of focused photograph, or photo taken at acute angle of the fin |
| 4 | Poorly focused, backlit or angled photograph taken at long distance to dolphin |

**Appendix 2.** Basic genetic indices for 15 microsatellite loci in the three populations identified by STRUCTURE and DAPC, *Coastal Shannon* (*n* = 54, including individuals sampled in Cork harbour), *Coastal mobile* (*n* = 25) and *Pelagic* (*n* = 18).

|  | *Coastal Shannon* | |  |  |  |  |  |  |  |  |
| --- | --- | --- | --- | --- | --- | --- | --- | --- | --- | --- |
|  | *N* | A | % | AR | H_O_ | H_E_ | HWE | *F*_IS_ | *F*_IS_ Low | *F*_IS_ High |
| *D18*^a^ | 54 | 3 | 33.3 | 2.2 | 0.150 | 0.140 | 1.000 | -0.071 | -0.124 | -0.027* |
| *D22*^a^ | 51 | 4 | 40.0 | 3.7 | 0.570 | 0.620 | 0.715 | 0.083 | -0.109 | 0.281 |
| *Dde*59^b^ | 52 | 4 | 50.0 | 4.0 | 0.790 | 0.710 | 0.893 | -0.116 | -0.261 | 0.040 |
| *Dde*61^b^ | 52 | 3 | 60.0 | 3.0 | 0.690 | 0.580 | 0.096 | -0.187 | -0.350 | -0.016* |
| *Dde*65^b^ | 51 | 4 | 66.7 | 3.6 | 0.530 | 0.450 | 0.108 | -0.167 | -0.277 | -0.043* |
| *Dde*66^b^ | 50 | 2 | 20.0 | 2.0 | 0.200 | 0.180 | 1.000 | -0.111 | -0.185 | -0.050* |
| *Dde*69^b^ | 54 | 4 | 80.0 | 4.0 | 0.610 | 0.540 | 0.810 | -0.136 | -0.264 | 0.016 |
| *Dde*72^b^ | 53 | 5 | 55.6 | 4.8 | 0.570 | 0.600 | 0.660 | 0.056 | -0.103 | 0.225 |
| *GATA098*^c^ | 52 | 4 | 44.4 | 4.0 | 0.730 | 0.700 | 0.468 | -0.037 | -0.182 | 0.119 |
| *Ttr*04^d^ | 54 | 3 | 37.5 | 3.0 | 0.700 | 0.620 | 0.654 | -0.141 | -0.318 | 0.046 |
| *Ttr*11^d^ | 53 | 3 | 30.0 | 3.0 | 0.640 | 0.580 | 0.637 | -0.113 | -0.293 | 0.079 |
| *Ttr*34^d^ | 53 | 4 | 66.7 | 3.2 | 0.640 | 0.550 | 0.134 | -0.174 | -0.402 | 0.056 |
| *Ttr*48^d^ | 54 | 4 | 57.1 | 4.0 | 0.700 | 0.640 | 0.875 | -0.093 | -0.242 | 0.068 |
| *Ttr*63^d^ | 54 | 9 | 52.9 | 7.6 | 0.910 | 0.850 | 0.784 | -0.070 | -0.157 | 0.030 |
| *Ttru*AAT44^e^ | 54 | 4 | 50.0 | 4.0 | 0.590 | 0.580 | 0.241 | -0.023 | -0.198 | 0.163 |
| Overall | 53 | 60 | 49.6 | 3.7 | 0.600 | 0.560 | 0.847 | -0.083 | -0.130 | -0.038* |

|  | *Coastal mobile* | |  |  |  |  |  |  |  |  |
| --- | --- | --- | --- | --- | --- | --- | --- | --- | --- | --- |
|  | *N* | A | % | AR | H_O_ | H_E_ | HWE | *F*_IS_ | *F*_IS_ Low | *F*_IS_ High |
| *D18*^a^ | 25 | 4 | 44.4 | 3.4 | 0.520 | 0.580 | 0.273 | 0.108 | -0.243 | 0.457 |
| *D22*^a^ | 24 | 4 | 40.0 | 3.5 | 0.500 | 0.560 | 0.198 | 0.101 | -0.259 | 0.468 |
| *Dde*59^b^ | 25 | 3 | 37.5 | 2.8 | 0.520 | 0.540 | 0.604 | 0.031 | -0.321 | 0.389 |
| *Dde*61^b^ | 25 | 3 | 60.0 | 2.9 | 0.280 | 0.390 | 0.089 | 0.283 | -0.092 | 0.657 |
| *Dde*65^b^ | 25 | 2 | 33.3 | 2.0 | 0.320 | 0.320 | 1.000 | 0.000 | -0.296 | 0.424 |
| *Dde*66^b^ | 25 | 2 | 20.0 | 1.5 | 0.000 | 0.080 | 0.020 | 1.000 | 1.000 | 1.000 |
| *Dde*69^b^ | 24 | 3 | 60.0 | 3.0 | 0.580 | 0.530 | 1.000 | -0.098 | -0.338 | 0.178 |
| *Dde*72^b^ | 25 | 4 | 44.4 | 3.8 | 0.480 | 0.570 | 0.029 | 0.152 | -0.141 | 0.464 |
| *GATA098*^c^ | 24 | 4 | 44.4 | 3.8 | 0.750 | 0.660 | 0.541 | -0.131 | -0.340 | 0.095 |
| *Ttr*04^d^ | 25 | 4 | 50.0 | 4.0 | 0.640 | 0.630 | 0.716 | -0.015 | -0.268 | 0.255 |
| *Ttr*11^d^ | 24 | 3 | 30.0 | 2.8 | 0.500 | 0.390 | 0.725 | -0.274 | -0.445 | -0.138 |
| *Ttr*34^d^ | 25 | 5 | 83.3 | 5.0 | 0.800 | 0.760 | 0.848 | -0.046 | -0.226 | 0.152 |
| *Ttr*48^d^ | 25 | 3 | 42.9 | 2.9 | 0.280 | 0.250 | 1.000 | -0.118 | -0.214 | -0.041 |
| *Ttr*63^d^ | 24 | 6 | 35.3 | 5.3 | 0.710 | 0.610 | 0.883 | -0.154 | -0.303 | 0.008 |
| *Ttru*AAT44^e^ | 25 | 4 | 50.0 | 3.8 | 0.520 | 0.630 | 0.291 | 0.170 | -0.093 | 0.450 |
| Overall | 25 | 54 | 45.0 | 3.4 | 0.490 | 0.500 | 0.361 | 0.013 | -0.066 | 0.088 |

|  | *Pelagic* | |  |  |  |  |  |  |  |  |
| --- | --- | --- | --- | --- | --- | --- | --- | --- | --- | --- |
|  | *N* | A | % | AR | H_O_ | H_E_ | HWE | *F*_IS_ | *F*_IS_ Low | *F*_IS_ High |
| *D18*^a^ | 18 | 8 | 88.9 | 7.5 | 0.890 | 0.840 | 0.827 | -0.055 | -0.193 | 0.120 |
| *D22*^a^ | 18 | 9 | 90.0 | 8.2 | 0.890 | 0.830 | 0.354 | -0.067 | -0.220 | 0.115 |
| *Dde*59^b^ | 18 | 8 | 100.0 | 7.1 | 0.720 | 0.820 | 0.294 | 0.120 | -0.156 | 0.413 |
| *Dde*61^b^ | 18 | 5 | 100.0 | 4.9 | 0.720 | 0.730 | 0.039 | 0.013 | -0.269 | 0.299 |
| *Dde*65^b^ | 18 | 5 | 83.3 | 4.7 | 0.890 | 0.750 | 0.856 | -0.180 | -0.336 | 0.012 |
| *Dde*66^b^ | 18 | 10 | 100.0 | 7.7 | 0.720 | 0.680 | 0.281 | -0.064 | -0.269 | 0.129 |
| *Dde*69^b^ | 17 | 5 | 100.0 | 4.9 | 0.820 | 0.750 | 0.476 | -0.104 | -0.352 | 0.157 |
| *Dde*72^b^ | 18 | 8 | 88.9 | 7.8 | 0.940 | 0.850 | 1.000 | -0.105 | -0.214 | 0.033 |
| *GATA098*^c^ | 18 | 8 | 88.9 | 7.0 | 0.830 | 0.770 | 0.362 | -0.084 | -0.294 | 0.136 |
| *Ttr*04^d^ | 18 | 8 | 100.0 | 7.4 | 0.830 | 0.830 | 0.346 | 0.000 | -0.187 | 0.201 |
| *Ttr*11^d^ | 18 | 10 | 100.0 | 8.4 | 0.890 | 0.820 | 0.881 | -0.079 | -0.215 | 0.076 |
| *Ttr*34^d^ | 17 | 6 | 100.0 | 5.5 | 0.710 | 0.760 | 0.843 | 0.077 | -0.174 | 0.351 |
| *Ttr*48^d^ | 17 | 7 | 100.0 | 6.5 | 0.820 | 0.810 | 0.136 | -0.022 | -0.224 | 0.201 |
| *Ttr*63^d^ | 18 | 14 | 82.4 | 10.6 | 0.830 | 0.830 | 0.567 | -0.002 | -0.167 | 0.188 |
| *Ttru*AAT44^e^ | 18 | 8 | 100.0 | 6.9 | 0.830 | 0.780 | 0.353 | -0.065 | -0.251 | 0.146 |
| Overall | 18 | 119 | 94.8 | 7.0 | 0.820 | 0.790 | 0.585 | -0.040 | -0.096 | 0.013 |

*N* = number of individuals, A = number of alleles observed, % = percentage of total alleles, AR = allelic richness, H_O_ = observed heterozygosity, HE = expected heterozygosity, are given as per locus per population sample. HWE = corrected *p*-values (chi-square test for goodness-of-fit), *F*_IS_ = *F*_IS_ values for each loci and population sample (overall), *F*_IS_ Low/High = bias corrected 0.025% and 92.5% percentiles of the confidence interval. *denotes significance in *F*_IS_ values as the 95% CI does not overlap zero.

^a^Shinohara et al. 1997.

^b^Coughlan et al. 2006.

^c^Palsbøll et al. 1997.

^d^Rosel et al. 2005.

^e^Caldwell et al. 2002.

**
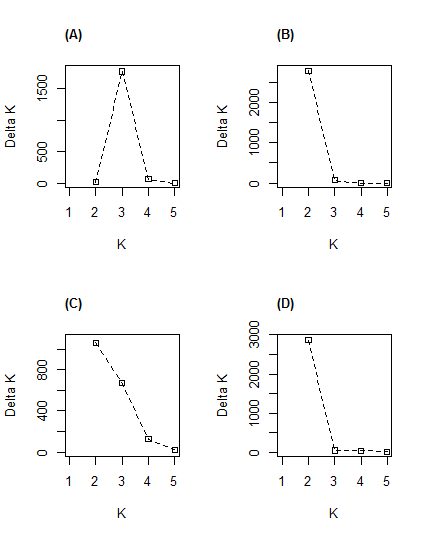
**

**Appendix 3.** Second order rate of change for mean log-likelihood, *∆K* (*i.e.* Evanno-method), for different number of populations, *K*, (A) including all samples, (B) only coastal samples (*Shannon* and *mobile*) included, (C) all samples included and close relatives (*r* ≥ 0.45) removed, and (D) only coastal samples included and close relatives removed.


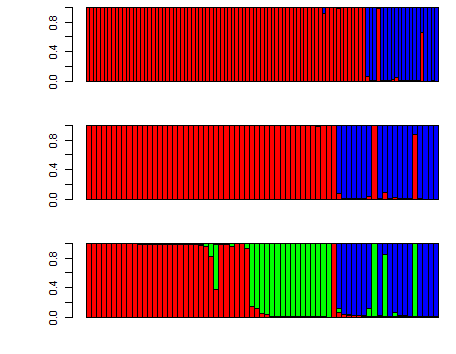


(B)

(C)

COASTAL BIOPSIES

STRANDED SAMPLES

‘bnd204’

(A)

COASTAL BIOPSIES

STRANDED SAMPLES

**Appendix 4.** Population assignment probabilities (STRUCTURE) of (A) all Irish bottlenose dolphins with *K* = 2, (B) after removal of close relatives (*r* ≥ 0.45) from the dataset with *K* = 2, and (C) after removal of close relatives (*r* ≥ 0.45) from the dataset with *K* = 3, where *K* is the candidate number of populations. Each vertical column corresponds to an individual dolphin. Note that sample ‘bnd204’ is assigned to ‘pelagic’ cluster at *K* = 2 (B) and to ‘coastal mobile’ cluster at *K* = 3 (C), when close relatives are removed.


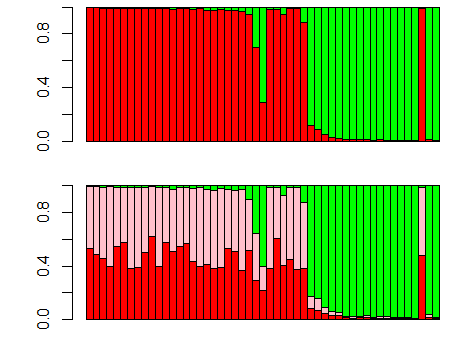


SHANNON BIOPSIES/STRANDINGS

BIOPSIED OUTSIDE SHANNON OR CORK

POSSIBLE MIGRANT

CORK BIOPSY

STRANDED OUTSIDE SHANNON

(A)

(B)

**Appendix 5.** Population assignment probabilities (STRUCTURE) of coastal Irish bottlenose dolphins (*Shannon* and *mobile*) after removal of close relatives (*r* ≥ 0.45) from the dataset, with (A) *K* = 2, and (B) *K* = 3, where K is the candidate number of populations. Each vertical column corresponds to an individual dolphin.


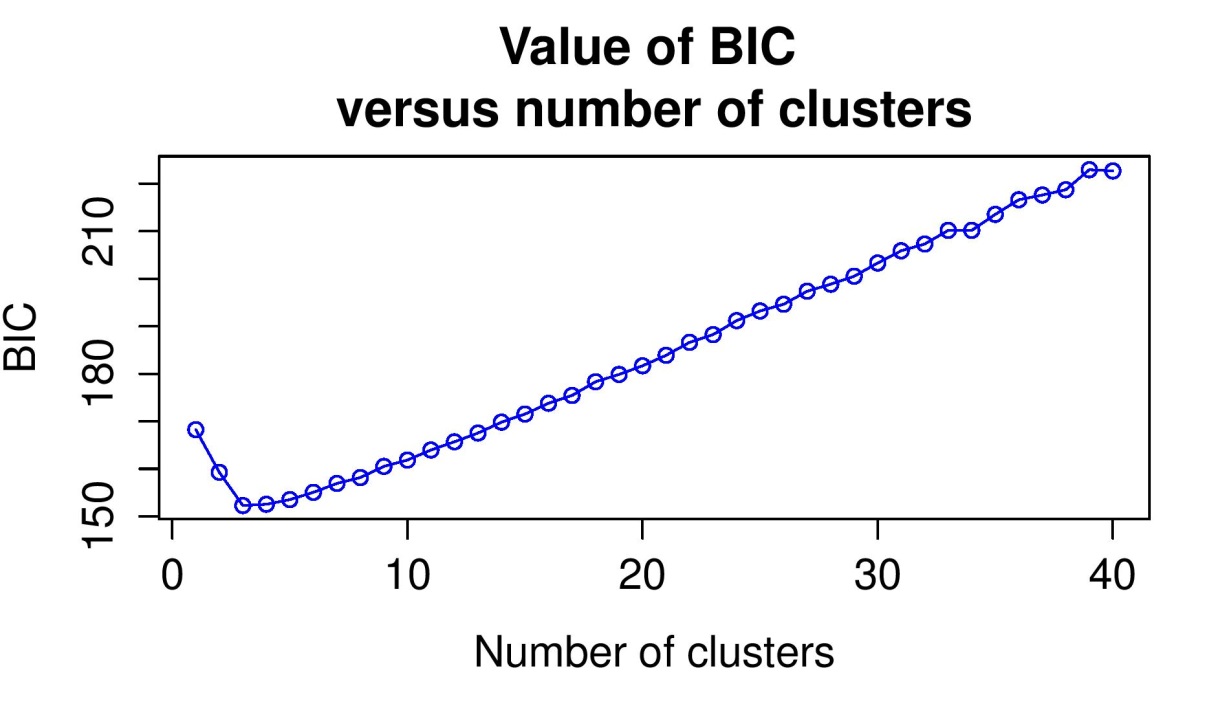


COASTAL BIOPSIES

STRANDED SAMPLES

**Appendix 6.** Bayesian Information Criterion (BIC) values plotted against the number of clusters. The lowest BIC-value indicates the most likely number of clusters (*K* = 3).


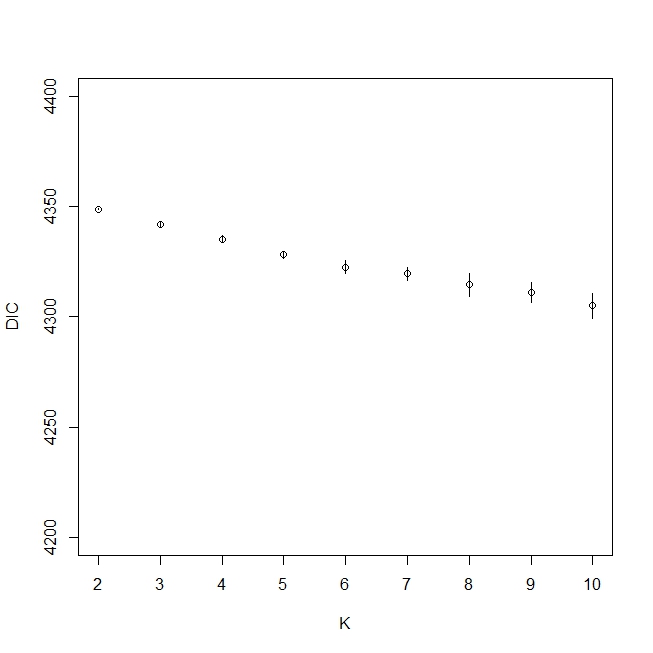


**Appendix 7.** Mean Deviance Information Criterion (DIC) values (with SD) using ten replicate TESS runs for each candidate number of populations (*K*) varying from 2 to 10.


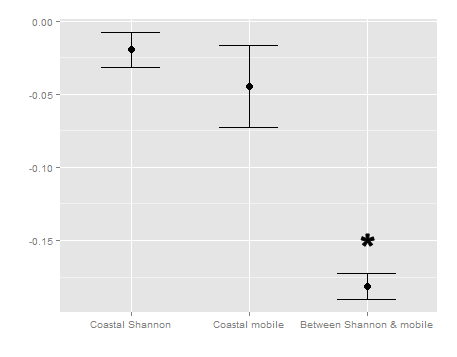


**Appendix 8.** Mean relatedness coefficient *r* (Queller & Goodnight 1989) with 95% confidence interval within and between the two bottlenose populations, *Coastal Shannon* and *Coastal mobile*. * denotes a significant difference with Kruskal-Wallis *p* < 0.0001.


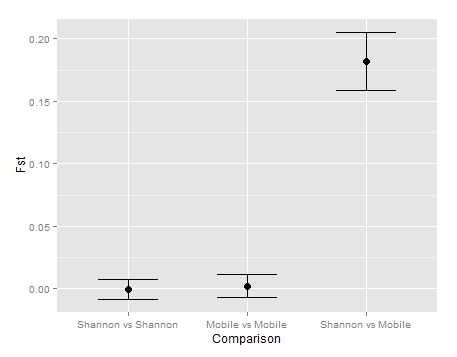


**Appendix 9.** Average F_ST_ values (with 95% CI) from ten runs with 20 randomly selected individuals from either *Coastal Shannon*, *Coastal mobile* or from both populations after dividing individuals randomly into two populations of ten.

**Appendix 10.** Indices used when testing for sex-biased dispersal and their corresponding *P*-values.

|  | *N* | Mean assignment | Var (assignment) | *F*_IS_ | *F*_ST_ | Relatedness | *H*_O_ | *H*_S_ |
| --- | --- | --- | --- | --- | --- | --- | --- | --- |
| Females | 31 | -1.167 | 14.328 | -0.039 | 0.161 | 0.286 | 0.550 | 0.530 |
| Males | 66 | 0.548 | 9.220 | -0.038 | 0.185 | 0.320 | 0.523 | 0.504 |
| P-value |  | 0.993 | 0.829 | 0.773 | 0.625 | 0.784 | 0.822 | 0.972 |

*N* = number of individuals, *F*_IS_ = *F*_IS_ value for each population sample (across all loci), *F*_ST_ = *F*_ST_ value for each population sample (across all loci), H_O_ = observed heterozygosity, H_S_ = observed gene diversity.

**Appendix 11.** Standardized Lagged Association Rate (SLAR), the probability that associates remain together divided by the mean number of associates, for bottlenose dolphins encountered ≥5 times (A) in the Shannon Estuary, and (B) outside Shannon Estuary in the coastal waters of Ireland, during the study period 1996-2014. SLAR is represented by the blue line, null association rate (expected if all individuals associate at random) by the red line, and the best fitting model by the green line (“casual acquaintances” in the *Coastal Shannon*, and “constant companions and casual acquaintances” in the *Coastal mobile* subset). Time lag (number of days) is given on logarithmic scale.

**Appendix 12.** Lagged Identification Rate (LIR) models (with their respective AIC/QAIC values) from Whitehead (2009) fitted to explain the probability to encounter individual bottlenose dolphins that had been encountered before within the Shannon estuary (*Coastal Shannon*) or outside of it (*Coastal mobile*).

| Area | Model | Model description | AIC/QAIC |
| --- | --- | --- | --- |
| *Coastal Shannon* | (a1) | Closed | 74504.7 |
|  | (a2*exp(-a1*td)) | Emigration/mortality | **74173.2** |
|  | (a2+a3*exp(-a1*td)) | Emigration+reimmigration | 74502.2 |
|  | (a3*exp(-a1*td)+a4*exp(-a2*td)) | Emigration+reimmigration+mortality | 74177.0 |
|  | (a1*cos(a2*td)+a3) | Seasonal emigration+reimmigration | 74508.7 |
|  |  |  |  |
| *Coastal mobile* | (a1) | Closed | 4190.4† |
|  | (a2*exp(-a1*td)) | Emigration/mortality | **4163.0**† |
|  | (a2+a3*exp(-a1*td)) | Closed: Emigration+reimmigration | 4177.0† |
|  | (a3*exp(-a1*td)+a4*exp(-a2*td)) | Emigration+reimmigration  +mortality | 4168.3† |
|  | (a1*cos(a2*td)+a3) | Seasonal emigration+reimmigration | 4193.9† |

a1 = emigration rate, a2 = mean time in study area, a3 = mean time out of study area, a4 = Mortality rate, td = time lag. † denotes QAIC-values for models with a quasi-distribution and variation inflation factor of 2.1.

**LITERATURE CITED**

Caldwell M, Gaines MS, Hughes CR (2002) Eight polymorphic microsatellite loci for bottlenose dolphin and other cetacean species. Mol Ecol Notes 2:393–395

Coughlan JL, Mirimin L, Dillane E, Rogan E, Cross T (2006) Isolation and characterization of novel microsatellite loci for the short-beaked common dolphin (*Delphinus delphis*) and cross-amplification in other cetacean species. Mol Ecol Notes 6:490–492

Englund A, Ingram S, Rogan E (2008) An updated population status report for bottlenose dolphins using the lower River Shannon SAC in 2008. Final Report to the National Parks and Wildlife Service, University College Cork, Ireland, 34 pp

Palsbøll PJ, Berube M, Larsen AH, Jørgensen H (1997) Primers for the amplification of tri- and tetramer microsatellite loci in baleen whales. Mol Ecol 6:893–895

Rosel PE, Forgetta V, Dewar K (2005) Isolation and characterization of twelve polymorphic microsatellite markers in bottlenose dolphins (*Tursiops truncatus*). Mol Ecol Notes 5:830–833

Shinohara M, Domingo-Roura X, Takenaka O (1997) Microsatellites in the bottlenose dolphin *Tusiops truncatus*. Mol Ecol 6:695–696

Whitehead H (2009) SOCPROG programs: Analyzing animal social structures. Behav Ecol Sociobiol 63:765–778
